# Detection and initial management of gestational diabetes through primary health care services in Morocco: An effectiveness-implementation trial

**DOI:** 10.1371/journal.pone.0209322

**Published:** 2018-12-28

**Authors:** Bettina Utz, Bouchra Assarag, Tom Smekens, Hassan Ennassiri, Touria Lekhal, Nawal El Ansari, Bouchra Fakhir, Amina Barkat, Amina Essolbi, Vincent De Brouwere

**Affiliations:** 1 Institute of Tropical Medicine, Antwerp, Belgium; 2 National School of Public Health, Rabat, Morocco; 3 Service des Réseaux des Etablissements de Santé al Haouz, Tahanaout, Morocco; 4 Service des Réseaux des Etablissements de Santé, Marrakech, Morocco; 5 Faculty of Medicine, Cadi Ayyad University, Marrakech, Morocco; 6 Faculty of Medicine and Pharmacy, Mohammed V University, Rabat, Morocco; TNO, NETHERLANDS

## Abstract

**Background:**

Gestational Diabetes Mellitus (GDM) testing and management in Morocco is associated with delays resulting in late commencement of treatment. To reduce delays and to increase access of women to GDM care, a country-adapted intervention targeting primary health care providers was designed to test the hypothesis that detection and initial management of GDM at the primary level of care improves newborn outcomes in terms of lower birthweights and less cases of macrosomia and impacts on maternal weight gain, glucose balance and pregnancy outcomes.

**Materials and methods:**

We conducted a cluster randomized controlled trial in two districts of Morocco. In each district, 10 health centers were randomly selected to serve either as intervention or control sites. Pregnant women attending antenatal care in the study facilities were eligible to participate. At the intervention sites, women were offered GDM screening by capillary glucose testing following International Association of Diabetes in Pregnancy Study Groups/WHO criteria. Women diagnosed with GDM received counselling on nutrition and exercise and were followed up through their health center whereas at control facilities routine practice was applied. Primary outcome was birthweight and secondary outcomes maternal weight gain, glucose control and pregnancy complications. We further assessed GDM prevalence in the intervention arm. Statistical analysis was performed on 210 recruited women. Continuous variables were reported using means while categorical variables using frequencies with tests of independence applying chi-squared tests. Differences of outcome variables between the two groups were estimated by mixed-effects regression models and effect sizes adjusted for confounders. The trial is registered under NCT02979756 at ClinicalTrials.gov.

**Results:**

GDM prevalence reached 23.7% in Marrakech. Birthweight in the intervention group was 147grams lower than in the control group (p = 0.08) as was the proportion of macrosomes (3.5% versus 18.4%; p< 0.001). In the intervention arm, women did two times more follow-ups than at control sites (p = 0.001) and mean follow-up intervals were shorter (11.3 days versus 18.7 days; p < 0.001). Overall, 30% more fasting blood sugar values were balanced (p = 0.005) and mean weekly maternal weight gain 49 grams lower (p = 0.032) in the intervention group. More women from control facilities had a delivery complication whereas more newborn complications were observed in women from intervention facilities. No difference between the two groups existed regarding mode of delivery and mean gestational age at delivery. One of the main limitations of the study was the Hawthorn-effect at control sites that might have led to an underestimation of the effect size.

**Conclusion:**

A high GDM prevalence in Morocco calls for a context-adapted screening and management approach to enable early interventions. GDM detection and care through antenatal care at primary health facilities may have positively impacted on newborn birthweight but findings are inconclusive. Results of this study will contribute to the decision on a potential upscaling of the intervention in Morocco. Future research could examine long term metabolic changes including diabetes type 2 in the cohort of women and their children.

## Introduction

Worldwide, every sixth woman is affected by hyperglycemia in pregnancy and low- and middle-income countries carry more than 90% of the global burden. Prevalence is highest in South East Asia (24.2%), and the Middle East and North African region are already second with prevalence rates reaching 21.8%, while in North America and Europe prevalence ranges between 14.6% and 16.2%.[1]

Timely detection of gestational diabetes (GDM) and initiation of treatment are important to interrupt the impact of maternal hyperglycemia on the fetal development and to reduce the negative immediate and lifelong consequences of fetal hyperinsulinemia.[2,3] Furthermore, screening in pregnancy provides an additional opportunity to prevent the development of a future diabetes in mothers and their offspring [4,5,6], particularly in settings where antenatal care (ANC) often remains the only contact of women with the health care sector.

Already proposed by O’Sullivan in 1973 [7], universal screening has been widely advocated in 2010 by the International Association of Diabetes in Pregnancy Study Groups (IADPSG) [8], given that selective screening misses out over 40% of affected women [9]. However, only recently did the International Federation of Gynecology and Obstetrics (FIGO) publish GDM consensus guidelines taking not only into consideration a universal screening approach but some of the obstacles to screening prevalent in low- and middle-income settings.[10]

In Morocco, GDM screening has not yet been widely adopted, and a situational analysis conducted in two districts revealed that laboratory tests as well as diagnosis and management of GDM were often associated with long delays resulting in either undiagnosed cases or in a diagnosis a posteriori after the occurrence of a complication.[11] To reduce delays and to increase access of women to screening and treatment, members of the gestational diabetes research group composed of specialists, researchers and representatives of the Ministry of Health Morocco came up with an intervention targeting first line health care providers who offer ANC in primary health care services. To test the hypothesis that detection and initial management of GDM at the primary level of care would reduce the incidence of macrosomia and result in lower birthweights of newborns, and to assess its potential for a future scaling up, we conceived this hybrid effectiveness-implementation research using a cluster randomized controlled trial design.

## Materials and methods

### Design and study population

As part of an effectiveness implementation study, we conducted a cluster randomized controlled trial in the region of Marrakech-Safi located in central Morocco and covering part of the Atlas Mountains. Two districts, Marrakech and Al Haouz were chosen for this implementation trial with the former being mainly urban and the latter being a predominantly rural district with limited geographical access to health care services. Around 1.95 million people live in the area, where 92 health centers and 53 dispensaries provide primary health care to the population.[12]

We chose as unit of randomization the health center (cluster) to limit contamination between the different arms. To select the health facilities for the study, we draw from the list of health centers provided by the district directorates all those that performed on average at least 30 monthly ANC consultations (new cases). These were then continuously numbered in each district and a random number generator used to select 10 health centers per district, the first five serving as intervention and the remaining five as control facilities. In the selected facilities, all pregnant women of consenting age attending ANC and being newly diagnosed with GDM were eligible to participate in the trial. Pregnant women with a known diabetes type 1 or 2 were excluded.

### Intervention

ANC providers of the intervention facilities (two providers per site) received a 1-day training on GDM screening and management. The training targeted the application of new GDM algorithms for screening and management that were based on the latest FIGO consensus recommendations [10], the preparation and conduct of a 75g oral glucose tolerance test (OGTT) and the provision of advice on nutrition and physical exercise. All ANC services at the intervention sites received the necessary equipment for GDM screening including glucometers, test-strips, 75g OGTT solutions as well as brochures and guidelines on nutrition for women affected by GDM that had been developed by the Moroccan Ministry of Health. From 14^th^ November 2016 (World Diabetes Day) onwards, women attending ANC at the intervention sites were offered GDM screening by capillary glucose testing. Applying the IADPSG and WHO criteria as adopted by FIGO in their latest consensus recommendations for the diagnosis of GDM [8,10,13], women tested positive for GDM at the intervention facilities either by a fasting blood glucose (FBG) before 24 weeks or by a 75g OGTT in the second trimester received nutritional counselling by the ANC nurse and were followed up weekly to twice monthly up to eight weeks post-partum through the health center [14]. Cut-off values for the diagnosis of GDM were 0.92–1.25g/l (5.1−6.9 mmol/l) for fasting glucose, or 1.8g/l (10 mmol/l) and above one hour, or between 1.53 and 1.99 g/l (8.5–11.0 mmol/l) two hours after ingestion of 75g glucose [10]. If during follow-up glucose values exceeded internationally recommended targets for fasting or postprandial values [10,15], women were sent for consultation to the diabetes referral center. At the control facilities, screening and initial management of GDM followed routine practice using national recommendations.[16] According to latest national guidelines, pregnant women detected with a fasting glucose value of 0.92g/l in the first trimester or at their first ANC visit should be diagnosed with a GDM without a repeat diagnostic test being required. If the obligatory first trimester glucose test was not done or normal, a 75g OGTT is advised between 24 and 28 week gestational age (GA) following the international IADPSG thresholds for diagnosis. Providers were asked to recruit every woman with GDM until they reached at least 8 GDM patients who consented to participate in the study. The last woman in the intervention arm was included on 6^th^ June 2017 and in the control arm on 14^th^ November 2017. The trial is registered under NCT02979756 at ClinicalTrials.gov and was first posted on 2 December 2016, two weeks after starting the enrollment of study participants due to administrative reasons. The authors confirm that all ongoing and related trials for this intervention are registered.

### Outcome variables

Primary outcome measure was the difference in mean birthweight between the two arms, while secondary outcomes were maternal weight gain and glucose levels during follow-up, pregnancy related outcomes including mode of delivery, presence or absence of obstetric (prolonged labor, pre/eclampsia, shoulder dystocia and other) or newborn (respiratory distress, hypoglycemia and other) complications and prevalence of GDM at the health centers using as numerator the number of women tested positive for GDM and as denominator the number of pregnant women tested. Maternal weight (pre-pregnancy weight as reported by the mother and measured during ANC/ follow-up visits) as well as information on glucose levels (measured at the health center or in the private sector) were documented by the treating nurse on a preformatted data collection form. Information on maternal and newborn outcomes (birthweight, complications during delivery, Apgar) relied on information provided by the ANC nurses and was verified for 91% (n = 191) of women in the respective hospital records. For home deliveries (5.2%), birthweight had to be estimated based on the weight of the newborn taken post-partum at the health center (between 1 and 26 days after delivery). Birthweight was estimated by two independent researchers using the 50^th^ percentile of the weight change nomograms developed by Paul et al. [17].

### Sample size

We investigated the hypothesis that the primary outcome, mean birthweight, differs in both groups. Using previous studies on birthweight differences of newborns of GDM affected mothers receiving different treatment schemes [18,19], we assumed that mean birthweight of newborns in the intervention group would be 300 grams lower than mean birthweight in the control group (3400 versus 3700 grams with a standard deviation of 500 grams), due to earlier detection and treatment of GDM. For our sample size calculation, we applied an intra-class correlation coefficient of 0.1 and assumed 20 equally sized clusters. Under 80% power and 5% alpha, this yielded a sample size of 75 GDM affected women per treatment arm, or 7.5 per health center, which we rounded up to 8. Based on a previously reported GDM prevalence of 8,2% in Morocco [20], we assumed that we would need an average cluster size of at least 90 women tested for GDM per health center to detect 8 women with a GDM.

### Data collection and entry

Individual patient data including age, gestity, parity, presence of previous obstetric risk factors, last menstrual period, weight and height, GDM test results and date of diagnosis, treatment, information on referral and follow-up (glucose test results, weight, treatment) as well as routine monthly facility data were collected by the ANC nurse at the health center and reported on pre-formatted data collection forms. The research coordinators of the two districts visited the health centers in 4–6 weekly intervals to collect the information and clarify any inconsistencies. Data on outcomes was collected at the health center and at the place of delivery. All data was anonymized and double entered into a preformatted excel file, cleaned and later converted into STATA version 14 and R version 3.4.2 for further analysis.

### Statistical methods

Statistical analysis was performed on all 210 recruited women irrespective of gestational age at diagnosis and their follow-up adherence based on the intention-to-treat principle. Preterm deliveries (gestational age below 37 weeks; n = 8) were excluded from birthweight analyses. Continuous variables were reported using means and standard deviations or standard errors while categorical variables described by frequencies and percentages.

Differences of outcome variables between the two groups were estimated using the mixed-effects regression model by applying a linear mixed model for continuous outcomes, logistic for categorical outcomes and Poisson to estimate number of balanced blood sugar values, using the total number of measurements per patient as an offset variable. Most models employed two levels of random effects, patients and health centers. The analyses of maternal weight gain and time interval between follow-up visits were based on disaggregated within-person measurements and employed three levels: within patients, between patients and between health centers. Effect sizes were adjusted for maternal age, gestity and residence. All models were estimated using Restricted Maximum Likelihood (REML); the logistic and Poisson models additionally employed the Laplace approximation. The use of random intercepts resulted in a compound symmetry correlation structure. Tests of independence based on two-by-two contingency tables were performed as chi-squared tests with Rao and Scott’s (1984) correction to account for clustering within health centers [21].

### Ethics approval and consent to participate

All women included in this study were asked for written consent to participate. They were free to decline or stop their participation at any time without any consequences for their care. The study was approved by the institutional review board of the Institute of Tropical Medicine (Reference 1086/16) and the Ethics Committee of the University Hospital (Registration B300201628508) in Antwerp, Belgium and by the Ethics Committee for Biomedical Research at Mohammed V University, Rabat (Dossier 83/16).

## Results

**Fig 1:**
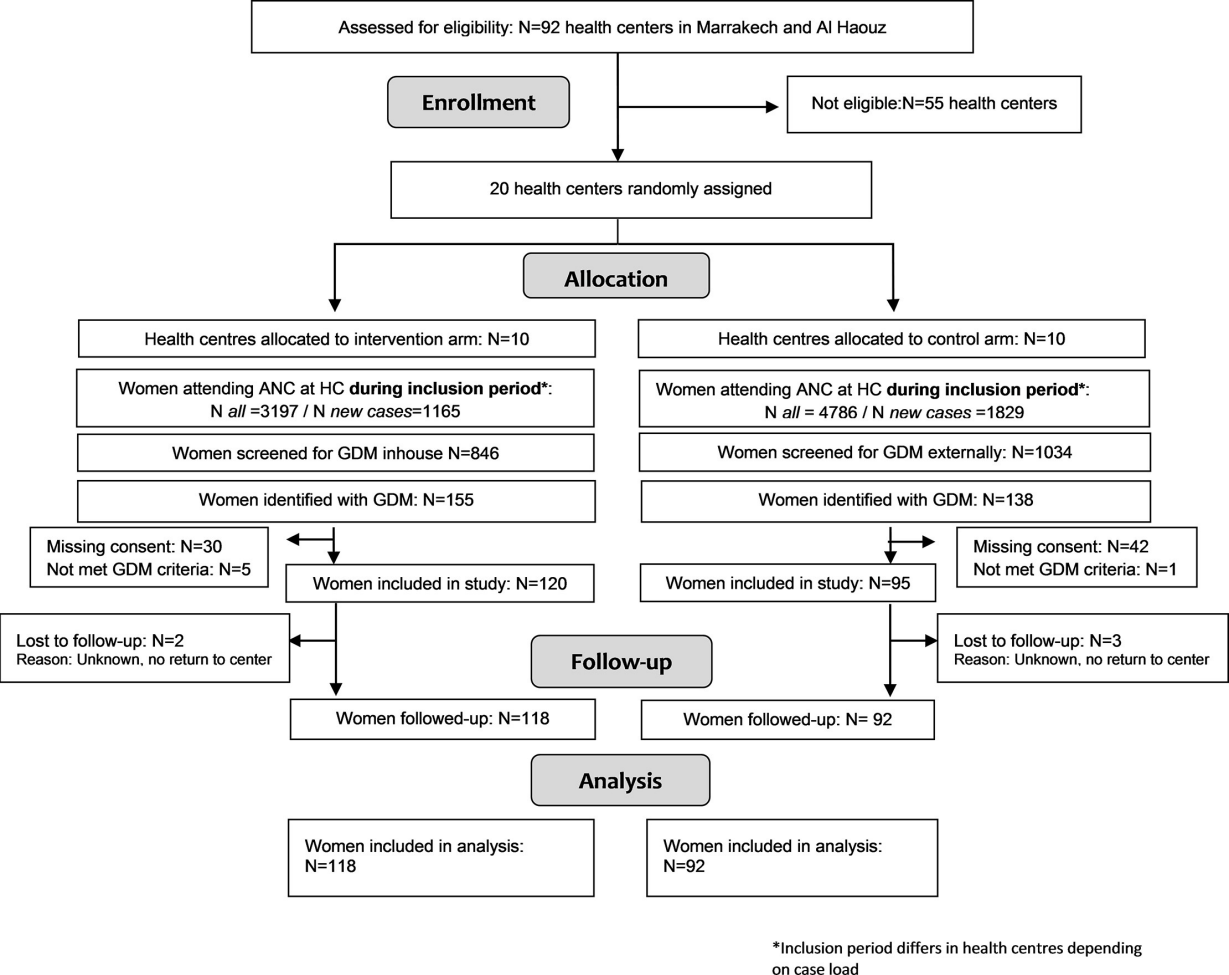
Consort trial flow chart.

Of 846 women screened at the intervention sites, 155 (18.3%) were eligible to participate in the trial. At the control facilities, 138 of 1034 (13.3%) women who presented with a glucose test result at antenatal care were eligible to participate in the study. At all study sites, a total of 215 (73.4%) women were included in the trial, 120 (77.4%) women at the intervention sites and 95 (68.8%) women at the control facilities. However, five women did not return to their health centers after the first encounter (lost to follow up), two in the intervention and three in the control sites (Fig 1). We included 210 (97.7%) women in the analysis, 118 (98.3%) women diagnosed at the intervention and 92 (96.8%) women at the control facilities.

Mean age of the women was 28 and 27 years respectively (see Table 1 for baseline characteristics). More women of the control sites had already a history of gestational diabetes, a delivery of a macrosomic or preterm baby and a medical history of hypertension. In the intervention group, more women were primigravidae compared to the control group. Although the majority of women in both arms came from rural areas, their proportion was higher in the control group. Women of the intervention facilities had a higher educational level than women of the control health centers.

**Table 1:** Baseline characteristics of the study population.

	Intervention group (n = 118)	Control group (n = 92)
**Mean age;** *years (SD)*	27.9 (6.5)	27.3 (6.7)
**History of;** *n (%)*		
Gestational diabetes	1 (0.85)	13 (14.1)
Hypertension	1 (0.85)	4 (4.3)
Abortion	25 (21.2)	21 (22.8)
Premature delivery	1 (0.85)	4 (4.3)
Stillbirth	6 (5.1)	4 (4.3)
Macrosomic baby	1 (0.85)	15 (16.3)
**Gestity;** *n (%)*		
G1	42 (35.6)	28 (30.4)
G2-3	53 (44.9)	42 (45.7)
G4+	23 (19.5)	22 (23.9)
**Residence;** *n (%)*		
Urban	55 (46.6)	28 (30.4)
Rural	63 (53.4)	64 (69.6)
**Distance to health center**^a^; *n (%)*		
<30 minutes	55 (46.6)	42 (45.7)
30–59 minutes	37 (31.4)	24 (26.1)
≥60 minutes	15 (12.7)	16 (17.4)
**Education**^a^; *n (%)*		
None	24 (20.3)	21 (22.8)
Primary	36 (30.5)	42 (45.7)
Secondary	38 (32.2)	16 (17.4)
Tertiary	9 (7.6)	3 (3.3)
**Socio-economic situation;** *n (%)*		
Housing^a^		
*Owning*	65 (55.1)	54 (58.7)
*Renting*	42 (35.6)	28 (30.4)
Personal transport^b^		
*None*	34 (28.8)	30 (32.6)
*Bike or moped*	58 (49.2)	35 (38)
*Car*	13 (11)	17 (18.5)
Health insurance coverage^a^	49 (41.5)	36 (39.1)

^a^ Information missing for 11 women in the intervention group and 10 women in the control group

^b^ Information missing for 13 women in the intervention group and 10 women in the control group

### Primary outcome

There was a difference in birthweight between the two groups, and birthweight in the intervention arm was 119 grams lower than in the control sites for all women irrespective of gestational age at birth. When excluding preterm deliveries (GA at delivery below 37 weeks), the difference was 147 grams. Table 2 presents the birthweight differences stratified by gestational age, blood sugar control and treatment. Findings indicate that the earlier GDM was detected, the larger the birthweight differences in the respective categories of gestational age at diagnosis (<24, 24–28,>28 weeks) between both groups. We further assessed the association between birthweight and glucose balance during follow-up and the differences in birthweight with regards to treatment. Newborns of women with a third or more fasting blood glucose values within the norm during follow-up had lower birthweights than newborns born to mothers with less than a third of balanced glucose values. Mean birthweight was higher in women on insulin treatment compared to women on diet (3431 versus 3341grams, p = 0.427; preterm deliveries excluded). However, birthweight differences between the study arms were highest in newborns of women who received diet, this effect diminished when comparing women on insulin treatment.

The incidence of macrosome babies, defined as newborns with a birthweight of 4000 and more grams was lower in the intervention group (3.5% versus 18.4% in the control arm; p<0.001). This difference also remained after accounting for previous history of delivering a big baby (for women without previous history, 3.5% versus 17.8%, p <0.001; for women with previous history, 0% versus 21%, p = 0.705). Mean birthweight of the few macrosome babies in the intervention group (n = 4) was 4500g, higher than the mean birthweight of macrosome newborns in the control group (4206g, n = 16; p = 0.056).

**Table 2:** Birthweight differences between intervention and control group.

	Intervention group *grams ± SD (n)*	Control group *grams ± SD (n)*	Adjusted intervention effect*^b^ *(CI)*	Adjusted p- value*
**Mean birthweight**^a^	3286 ± 433 (115)	3430 ± 512 (87)	-147.07 (-313.4 to 19.3)	0.08
**By GA at diagnosis**				
<24weeks	3275 ± 513 (37)	3479 ± 550 (34)	-227.96 (-582.4 to 126.5)	0.192
24–28 weeks	3321 ± 391 (48)	3457 ± 423 (21)	-134.54 (-373 to 103.9)	0.249
>28 weeks	3243 ± 397 (30)	3359 ± 529 (32)	-126.39 (-412 to 159.2)	0.361
**By % FBG within the norm at follow-up**				
<1/3	3678 ± 779 (9)	3525 ± 529 (28)	32.45 (-566.7 to 631.6)	0.907
1/3-2/3	3342 ± 454 (19)	3421 ± 585 (29)	-109.48 (-512.1 to 293.2)	0.567
>2/3	3233 ± 359 (87)	3350 ± 412 (30)	-106.00 (-277.9 to 65.9)	0.210
**By treatment**				
Diet only	3270 ± 393	3427 ± 520	-155.38 (-326.2 to 15.4)	0.072
Insulin	3415 ± 677	3500 ± 173	-26.88 (-1287.2 to 1233.4)	0.960

GA = gestational age; FBG = fasting blood glucose; SD = standard deviation; CI = confidence interval

*Adjusted for: maternal age, gestity, and residence

^a^ Preterm deliveries (delivered <37 weeks GA) excluded (n = 8)

^b^ Restricted maximum likelihood estimates

### Secondary outcomes

Secondary outcomes relate to screening, follow-up, delivery and the postpartum and are presented in Tables 3–5. Women in the intervention arm were detected on average 10 days earlier than women in the control group. Most women in the intervention group were diagnosed between 24 and 28 weeks gestational age whereas in the control group most women were diagnosed before 24 weeks GA, although a substantial proportion (35.8%) was diagnosed later than 28 weeks GA. In the intervention sites, the diagnosis was based on a positive FBG in 62% of the women, whereas 38.2% were detected with a 75g OGTT. Treatment for the majority of women consisted of diet (91.4%), whereas 8.6% received insulin, 11.9% of women in the intervention and 4.3% of women in the control group. Slightly more women in the intervention arm were referred to a specialist (usually to a public diabetes referral centre) compared to the control sites, but 37% less women from the intervention facilities complied with the referral. Overall, women of the intervention group did on average 7.5 follow-ups, of which 6.7 visits at their health centre compared to 3.8 follow-up visits in the control arm, of which 3.2 visits at their health centre. The mean follow-up interval at the intervention sites was 11.3 days compared to 18.7 days at the control facilities (p<0.001). There was no difference in mean fasting blood sugar results at follow-up between intervention (0.87g/l) and control sites (0.88g/l). The rate of FBG measurements within the norm was 30% higher in the intervention group compared to the control group. Proportions of postprandial glucose values within the norm were similar between both groups although the number of measurements taken at the control sites was too low to draw statistical conclusions. Mean weekly maternal weight gain was 49 grams lower in women from the intervention sites (p = 0.032).

**Table 3:** Information on screening, treatment and follow-up outcomes.

	Intervention group (n = 118)	Control group (n = 92)	Adjusted intervention effect*^#^ *(CI)*	Adjusted p-value*
***Screening***				
**Average gestational age at GDM diagnosis***; weeks (SD)*	24.3 (7.1)	25.7 (7.1)	-2.46 (-5.76 to 0.85)	0.136
**By category***; n (%)*				
<24 weeks	39 (33.1)	36 (39.1)		
24–28 weeks	48 (40.7)	23 (25.0)		
29–32 weeks	20 (17.0)	12 (13.0)		
≥33 weeks	11(9.3)	21(22.8)		
**Diagnosis based on;** *n (%)*				
FBG	73 (61.9)	92 (100)		
OGTT 1-hour	31 (26.3)	-		
OGTT 2-hour	14 (11.9)	-		
**Diagnostic glucose levels**				
FBG at diagnostic*; mean g/l (SD)*	0.99 (0.05)	0.99 (0.07)	-0.001 (-0.02 to 0.02)	0.913
OGTT 1-hr diagnostic*; mean g/l (SD)*	2.04 (0.3)	-		
OGTT 2-hr diagnostic*; mean g/l (SD)*	1.66 (0.12)	-		
***Treatment & Referral***				
**Treatment**				
Insulin (second line treatment)^a^*; n (%)*	14 (11.9)	4 (4.3)	3.10 (0.80 to 14.44)	0.093
**Referral to a specialist***; n (%)*	60 (50.8)	43 (46.7)	1.68 (0.22 to 16.66)	0.595
**Compliance with referral**^b^*; n (%)*			0.70 (0.17 to 3.04)	0.595
Yes	33 (55)	27 (62.8)		
No	26 (43.3)	13 (30.2)		
***Follow-up***				
**Follow-up visits**; *mean no*. *(SD)*				
All	7.5 (4.9)	3.8 (3.3)	3.82 (1.72 to 5.93)	0.001
At health center	6.7 (4.3)	3.2 (2.6)		
At referral center	0.8 (1.6)	0.7 (1.3)		
**FBG at follow-up within the norm**; *n (%)*			1.29 (1.10 to 1.58)^c^	0.005
<1/3 of all values	9 (7.6)	30 (32.6)		
1/3-2/3 of all values	21 (17.8)	30 (32.6)		
>2/3 of all values	88 (74.6)	32 (34.8)		
**Post-prandial blood glucose at follow-up within the norm**; *n (%)*				
<1/3 of all values	14 (12.1)	4 (15.4)		
1/3-2/3 of all values	30 (25.9)	7 (26.9)		
>2/3 of all values	72 (62.1)	15 (57.7)		
**Maternal weight gain/week***;mean grams (SE*^d^*)*	202.2 (13.2)	250.0 (18.5)	-48.91 (-93.77 to -4.04)	0.032

FBG = fasting blood glucose, OGTT: oral glucose tolerance test (75g); SD = standard deviation; SE = standard error; CI = confidence interval

*Adjusted for: maternal age, gestity, and residence

^#^ Intervention effects presented for continuous data as difference in means; for categorical data as odds ratios except for ^c^

^a^ Diet in all women started as first line treatment

^b^ For 4 women compliance is unknown

^c^ Poisson model using incidence rate ratio

^d^ Weekly weight gain estimated as regression coefficients (SE reported)

**Table 4:** Delivery outcomes.

	Intervention group (n = 118)	Control group (n = 92)	Adjusted intervention effect*^#^ *(CI)*	Adjusted p-value*
**Gestational age at delivery***; weeks (SD)*	40.01 (1.74)	40.01 (2.06)	-0.04 (-0.60 to 0.52)	0.883
**By category***; n (%)*				
<37 weeks	3 (2.5)	5 (5.4)		
37–39 weeks	39 (33.1)	24 (26.1)		
40–42 weeks	70 (59.3)	56 (60.9)		
≥43 weeks	6 (5.1)	7 (7.6)		
**Mode of delivery***; n(%)*			0.72 (0.27 to 1.81)	0.458
Vaginal delivery	94 (79.7)	72 (78.3)		
Caesarean section	24 (20.3)	20 (21.7)		
**By place***; n (%)*				
Health center maternity	26 (22)	33 (35.9)		
Regional/provincial hospital	27 (22.9)	14 (15.2)		
University hospital	51 (43.2)	32 (34.8)		
Private clinic	9 (7.6)	7 (7.6)		
Home	5 (4.2)	6^a^ (6.5)		
**Delivery complications**^b^*; n (%)*			0.37 (0.06 to 1.40)	0.151
Yes	9 (7.6)	14 (15.2)		
No	102 (86.4)	73 (79.3)		
**By type***; n (%)*				
Prolonged labor	5 (4.2)	4 (4.3)		
Shoulder dystocia	-	4 (4.3)		
Hypertensive disorder	2 (1.7)	2 (2.2)		
Hemorrhage	2 (1.7)	2 (2.2)		
Abruptio	-	1 (1.1)		
Placenta retention	-	1 (1.1)		
**Newborn complications**^c^*; n (%)*			1.92 (0.77 to 5.29)	0.179
Yes	16 (13.6)	7 (7.6)		
No	100 (84.8)	79 (85.9)		
**By type***; n (%)*				
Respiratory distress	7 (46.7)	6 (85.7)		
Hypoglycemia	2 (13.3)	-		
Prematurity	1 (6.7)	1 (14.3)		
Stillbirth^d^	5 (33.3)	-		

SD = standard deviation; CI = confidence interval

*Adjusted for: maternal age, gestity, and residence

^#^ Intervention effects presented for continuous data as difference in means; for categorical data as odds ratios

^a^ One delivery on the way to the health facility

^b^ No information available for 12 women (7 intervention, 5 control group)

^c^ No information available for 8 newborns (2 intervention, 6 control group)

^d^ Fresh stillbirths in 2 women with uncomplicated follow-up referred at 39 weeks GA to the university hospital for delivery; a third woman with uncomplicated follow-up sent at 40 weeks GA to deliver at the hospital but did not comply; delivered 3 weeks later. A macerated stillbirth was delivered at 40 weeks GA by a woman followed-up directly at reference center level. Another stillbirth occurred in a woman diagnosed with GDM at 30 weeks GA who after two uncomplicated follow-up visits presented at 34 weeks with oligohydramnios and yellow discharge and was referred to the university hospital.

**Table 5:** Postpartum follow-up.

	Intervention group (n = 118)	Control group (n = 92)	Adjusted intervention effect*^#^ *(CI)*	Adjusted p-value*
**Average interval between birth and glucose control postpartum***; days (SD)*	61.4 (27.8)	26.8 (24.3)	33.45 (20.76 to 46.14)	<0.001
**Post-partum glucose control**; *n (%)*	110 (93.2)	79 (86.8)	2.62 (0.80 to 8.62)	0.081
**By type***; n (%)*				
FBG	110 (93.2)	76 (82.6)		
OGTT^a^	105 (89.0)	-		
GPP	1 (0.8)	5 (5.4)		
**Mean glucose values at postpartum follow-up***; g/l (SD)*				
FBG	0.92 (0.13)	0.94 (0.15)	-0.02 (-0.09 to 0.05)	0.480
OGTT (2hours)	1.29 (0.30)	-		
**Postpartum value indicative of DM**^b^	4 (3.6)	4 (5.1)	0.72 (0.16 to 3.30)	0.659
**Based on**; *n (%)*				
FBG	2 (50.0)	4 (100)		
OGTT (2hours)	2 (50.0)	-		

FBG: fasting blood glucose; GPP: glucose post-prandial; DM: diabetes mellitus; SD = standard deviation; CI = confidence interval

*Adjusted for: maternal age, gestity, and residence

^#^ Intervention effects presented for continuous data as difference in means; for categorical data as odds ratios

^a^ All women who underwent an OGTT did also a FBG. Of the 105 women who underwent a post-partum OGTT, 5 did only a 1hour OGTT 4 women did not want to wait for 2 hours and 1 OGTT was stopped by the nurse after 1 hour as OGTT value was above 2g/l

^b^ Of 189 women tested postpartum; DM if FBG ≥1.26g/l or OGTT 2hours/GPP ≥2g/l

There was no difference in gestational age at delivery and the majority of women delivered between 40 to 42 weeks GA (calculation based on last menstrual period). However, a higher proportion of women in the intervention group delivered at a GA between 37 and 39 weeks compared to the control group (33.1% versus 26.1%). Most women at the intervention sites delivered at the university hospital (43.2%) whereas 35.9% of women of the control sites delivered at health centres with attached maternities. No difference existed between the groups in the proportion of women delivered by caesarean section (20.3% and 21.7% respectively). More women from control sites had delivery complications with prolonged labour and shoulder dystocia being the leading complications, whereas more newborns from mothers followed-up at intervention facilities experienced complications. Respiratory distress was the main complication of newborns. Five stillbirths were reported in women from intervention facilities and led to a separate review of each case by the study monitoring committee. None of the perinatal deaths were directly linkable to the care received at the health facility and related to various other external factors (care at referral centre, non-compliance, other obstetric problem). The highest proportion of newborns with a complication was found in the group of women detected at a GA below 24 weeks (n = 11; 47.8%). Of all women with a history of previous GDM (n = 14), 35.7% had a documented delivery complication compared to 64.3% of women without a previous episode of GDM (p = 0.009).

In the intervention arm women were told to return for retesting 6 to 8 weeks after delivery when vaccination of their baby was due. The average interval between delivery and post-partum testing in the intervention arm was 61 days compared to 27 days at the control sites. Fasting blood sugar was on average 0.92g/l in the intervention compared to 0.94g/l in the control arm. A blood glucose indicative of a diabetes mellitus (≥1.26g/l) was measured in eight of all women tested post-partum (4.2%). In the intervention arm, where FBG and 2-hour OGTT were routinely performed postpartum, two women had an OGTT result of ≥2.0g/l. These women would have gone undetected if only the fasting result would have been considered (Table 5).

### Prevalence of GDM

We calculated the prevalence of GDM at the intervention facilities (Table 6). Calculations were limited to the complete months of December 2016 until March 2017 when screening took place at all 10 intervention facilities. Prevalence calculations were based on at least one positive test result (either a FBG of ≥0.92g/l, a 1-hour 75g OGTT result of ≥1.80g/l or a 2-hour 75g OGTT result of ≥1.53g/l) following the screening algorithms [11]. Average GDM prevalence was significantly higher in Marrakech prefecture (23.7%) than in the more rural district of Al Haouz (18.3%, p = 0.047). The diagnosis of about two thirds of the patients was based on a positive fasting glucose value, while a third of women were diagnosed with a positive 1 or 2-hour OGTT measurement. Emesis as side effect after ingestion of 75g dextrose occurred in 1.98% of all women tested with an OGTT (n = 455).

**Table 6:** GDM prevalence in the intervention facilities between December 2016 and March 2017.

	Marrakech					Al Haouz					
Month	12.2016	1.2017	2.2017	3.2017	Mean	12.2016	1.2017	2.2017	3.2017	Mean	p-value
**No. of women tested**	162	164	97	156		109	110	75	94		0.047
**% of women tested positive**	21.6	23.8	26.8	23.7	23.7	20.2	19.1	24	10.6	18.3	
**% diagnosed with FBG**^1^	71.4	71.8	69.2	67.6	70.1	68.2	61.9	72.2	60.0	66.2	
**% diagnosed with OGTT**^2^	28.6	28.2	30.8	32.4	29.9	31.8	38.1	27.8	40.0	33.8	

^1^FBG: fasting blood glucose

^2^OGTT; oral glucose tolerance test

## Discussion

The findings of this research show lower birthweights in women who were detected and managed at intervention health centers although evidence was inconclusive. Nevertheless, there was a higher incidence of macrosomia in the control arm which may be considered a protective effect of the intervention. The impact of treatment on the reduction of macrosomia has been described in other studies.[22] We found a high prevalence of gestational diabetes with every fifth woman affected by GDM, although prevalence in rural was lower compared to urban areas. However, GDM prevalence is much higher than the prevalence of 8.2% measured in an earlier study conducted in Morocco [20], but goes in line with 21.8% prevalence rates reported for the Middle East and North African Region [23].

Two thirds of patients tested at the intervention sites were already diagnosed with GDM based on an elevated fasting blood sugar, while a third of women were diagnosed with an OGTT. This has implications for planning, as the majority of pregnant women attending ANC could already benefit from a fasting capillary glucose test at their health center. This could reduce costs of screening programs, as not all women would need to proceed to an OGTT. Furthermore, glucometers and test strips are already dispatched to primary health care facilities in the context of the chronic disease program, although their use is restricted to monitor patients with type 1 or 2 diabetes and to selectively screen for diabetes in individuals at high risk, but not to universally screen pregnant women in ANC.

Testing at intervention health centers enabled earlier diagnosis of GDM, and the mean difference between control and intervention settings in our study was ten days. Riskin-Mashiah et al. [24] demonstrated that already early pregnancy hyperglycemia impacts on neonatal outcomes. While outcomes are linked to the level of hyperglycemia [2], earlier detection and treatment nevertheless reduce exposure time of the unborn child to a hyperglycemic intrauterine environment. Our data showed that the earlier diagnosis and GDM management started, the larger the birthweight differences between control and intervention arm, underlining the importance of early active management of GDM.

Women at the intervention sites had on average seven follow-up visits at their health center compared to only three visits of women from control sites. Frequent contacts could have contributed to the intervention effects of lower birthweights, less maternal weight gain and better glucose control in the intervention group. Since compliance to referral was lower in women from intervention facilities, this may indicate that women perceived a referral providing low added value to the services at their health facility. Although our hypothesis of a birthweight difference of 300 grams between control and intervention group was too ambitious, the mean difference of 147 grams is in line with a treatment effect of 145 grams shown by Crowther et al. [3] in the ACHOIS trial.

Recommendations of the Institute of Medicine advise an average weekly pregnancy weight gain of 400 grams for women with a normal pre-pregnancy weight and 300 grams for overweight women.[25] The average weekly weight gain in both groups of our study population was below these recommended levels. Taking into account the increasing problem of obesity in Morocco with already 48% of women in reproductive age being overweight or obese [26], weight recommendations need to be re-considered. Feig and Naylor [27] critically pointed out the risks of current high thresholds and suggested an average weekly gain of between 170 to 285 grams over the course of a pregnancy. Other studies even suggested moderate weight loss in overweight and obese pregnant women to reduce maternal and neonatal adverse outcomes.[28]

A higher proportion of women from the intervention sites delivered at the regional or university hospitals compared to women of the control sites, of whom nearly 36% delivered at health center maternities. Although the proportion of reported delivery complications in the control arm were double compared to the intervention arm, more neonatal complications occurred in newborns of women from intervention facilities. Findings indicate the need to review the referral system for GDM that would furthermore demand a more adapted grading for its management. While women with a well-balanced GDM and without obstetric indications do not require giving birth at higher levels of care, referral could be limited to women on insulin, with an unbalanced GDM and those with obstetric indications. This requires a good integration of all three levels of care.

This is the first study that explores GDM prevalence and the effect of a GDM screening and management intervention on maternal and newborn outcomes at the first level of care in Morocco. The advantage of this implementation study is its integration into daily routine of ANC providers and as such, findings relate to reality at a primary health care setting. Despite the existing challenges for data collection prevalent in many low resource settings, through the involvement of on-site providers in prospective data collection and regular supervision visits, sufficient information could be gathered on a topic that has so far not received enough attention in Morocco. Particularly the scarcity of information about GDM at the primary level of care renders the results of this intervention important for national decision makers and contributes to a better understanding of the health impact of an intervention that has potential to be added to the existing package of ANC.

We applied the intention to treat approach and included all patients, whether diagnosed late or early, whether diet was well followed or not irrespective of the number of follow-ups to reflect the effectiveness of the intervention in real conditions. Nevertheless, the study has some limitations and therefore outcomes of the trial should be interpreted with caution. Women in the trial arms differed in some baseline characteristics such as a higher proportion of women with a previous history of GDM in the control arm. This imbalance could be an indicator of selection bias since providers in the control sites might still be used to selectively screening women with GDM based on risk factors such as previous GDM or macrosomic deliveries as advised in earlier national guidelines. This could have been avoided by excluding these patients from entering the study. Although we accounted for factors such as gestational age at inclusion, age and gestity in the analysis stage, there are other contextual factors such as family and husband support, motivation to adhere to treatment as well as access issues that may have influenced the measured outcomes. Regarding the primary outcome birthweight, one main limitation is the lack of comparable data on birthweight of newborns born to mothers not affected by GDM in the respective facilities. Data on birthweights of 5000 uncomplicated births collected at the University hospital of Rabat revealed average weights between 3229 and 3359 grams, indicating that mean birthweights in our sample corresponded to nationally comparable data [29]. Birthweight measurements were not directly observed and relied on reported data. Weighing scales in the different facilities differed and measurement errors due to calibration issues or human error may have occurred unnoticed. Pre-pregnancy weight of women was generally not available and weighing took place during ANC or follow-up visits, although measurements were not always taken at each visit. Nurses used as device the weighing scales available at their facilities, therefore measurements could be influenced by calibration errors and inter-device variations. However, this will not influence individual measurement differences as the same device was used over time. Gestational age calculation relied on the last menstrual period reported by the women and could be subject to recall bias. However, ANC nurses made substantial efforts to get information about the exact date and also used early ultrasound scans where available for their calculations. Compared to data collected during a pre-trial baseline assessment, we observed that the number of GDM cases diagnosed in the control facilities increased during the course of the study. An explanation for the observed changes would be the Hawthorne effect, indicating that providers changed their behavior as a response to being observed. Although the extent of such an effect remains difficult to assess [30], it may have led to an underestimation of the real effect of the intervention.

To reduce reporting error regarding birthweights, we tried to assure internal validity of the results by verifying documentation at the place of delivery. Validity of the glucose values was assured by using at all intervention facilities the same device with regular calibration by a code chip that comes with every new package of test strips. To assure that data is reliable, we regularly visited all the health centers and clarified any conflicting information with the facility providers. Despite our efforts to assure fidelity to the intervention design, results may not be purely attributable to the intervention, and other contextual factors such as individual provider motivation and workload during screening and counseling as well as the support patients received through their families may have played a role that were not taken into account by our methodology. However, since this was a new strategy integrated into an existing health care system and not a standalone intervention, we made sure that all changes were well documented as these reflect the challenges and opportunities of implementing a new activity to a real-life setting.

The study has been tailored to the primary health care level in Morocco but as we relied on the latest GDM consensus guidelines for GDM diagnosis [10], some of the results can be generalized to other countries with a similar context. Given the high prevalence of GDM in Morocco, findings of this study are relevant for further decisions on a potential regional or national upscaling of the intervention. However, the results also revealed that without a good coordination of the different health care levels involved, achievements at the primary level of care can be undermined.

Future research should assess individual components of the intervention and look at their separate impact on maternal and neonatal indicators. As the trial assessed immediate health outcomes, future cohort studies could examine on a longer-term recurrence of GDM and incidence of type 2 diabetes in these women. Furthermore, there is a need to examine the outcomes of newborns born to mothers in the two groups in terms of impact of the intervention on their metabolic status.

## Conclusions

GDM screening and its management at the primary level of care may have positively impacted on neonatal birth weight and maternal weight gain but findings are inconclusive. Results of this study indicate that GDM prevalence in Moroccan pregnant women is high and would require a screening and management approach that enables early interventions through the primary level of care.

## Supporting information

S1 File CONSORT checklist. (DOC)

S2 File Study protocol French. (PDF)

S3 File Study protocol English. (PDF)

## Abbreviations

ANC: Antenatal Care
FBG: Fasting Blood Glucose
FIGO: International Federation of Gynecology and Obstetrics
GA: Gestational Age
GDM: Gestational Diabetes Mellitus
IADPSG: International Association of Diabetes in Pregnancy Study Groups
OGTT: Oral Glucose Tolerance Test (OGTT)
