# Supplementary material for: Detection and initial management of gestational diabetes through primary health care services in Morocco: An effectiveness-implementation trial
Source: PLoS One. 2018 Dec 28;13(12):e0209322. doi: 10.1371/journal.pone.0209322 (PMC6310282; doi:10.1371/journal.pone.0209322)
Supplement: S2 File — (PDF) [file pone.0209322.s002.pdf]

Améliorer la détection et la prise en charge du diabète gestationnel au niveau des établissements de soins de santé primaires au Maroc

**Coordinating institution**	Ecole de Santé Publique (ENSP), Rabat, Maroc
**Investigators:**	Bettina Utz<br>Bouchra Assarag
**Promoter:**	Vincent De Brouwere
**Protocol Number:**	1
**Title:**	Améliorer la détection et la prise en charge du diabète gestationnel au niveau des établissements de soins de santé primaires au Maroc
**Version:**	1.0, Date 23.03.2016
**Coordinating institution:**	ENSP, Maroc
**Coordinating Investigator at ITM:**	Bettina Utz
**Department:**	Maternal and Reproductive Health Unit<br>Public Health Department
**Address:**	Institute of Tropical Medicine, Nationalestraat 155, 2000 Antwerp, Belgium
**Telephone/Fax:**	+32 3 247 6644/ +32 3 247 62 58
**Email:**	butz@itg.be

Statement of Compliance & Confidentiality

The information contained in this study protocol is privileged and confidential. As such, it may not be disclosed unless specific permission is given in writing by the ITM or when such disclosure is required by federal or other laws or regulations. These restrictions on disclosure will apply equally to all future information supplied which is privileged or confidential.

Once the final protocol has been issued and signed by the Investigator(s) and the authorized signatories, it cannot be informally altered. Protocol amendments have the same legal status and must pass through the mandatory steps of review and approval before being implemented.

By signing this document, the Investigator commits to carry out the study in compliance with the protocol, the applicable ethical guidelines like the Declaration of Helsinki and consistent with international scientific standards as well as all applicable regulatory requirements. The Investigator will also make every reasonable effort to complete the study within the timelines designated.

Investigators:

Bettina Utz Date: 23/03/2016

Bouchra Assarag:

Promotors:

Prof. Vincent De Brouwere

Contenu

Statement of Compliance & Confidentiality 2
Résumé 4
Contexte 5
Projet de recherche proposé 7
Objectifs et hypothèses 7
  Objectif général 7
  Hypothèses 7
  Objectifs spécifiques 8
Méthodes 9
  Devis de la recherche 9
  Durée 9
  Sites 9
  Population 11
  Stratégie d'échantillonnage 11
  Déroulement des différents volets 12
Evaluation 14
Collecte de données et outils 17
Analyse des données 19
Surveillance, supervision et contrôle de la qualité des données 19
Assurance qualité 19
Considérations éthiques 20
  Obtention du consentement éclairé 20
  Sécurisation des données 20
  Risques 20
  Avantages 21
  Utilisation des données de l'étude dans le cadre d'autres recherches 21
Dissémination des résultats 21
Calendrier 21
Budget 22
Références 23

Résumé

Le diabète gestationnel (DG) et le diabète préexistant sont associés à un certain nombre de complications chez la mère et son nouveau-né, lesquelles sont la pré-éclampsie, les accouchements prématurés et la mortinatalité. Dans les pays à revenu faible et moyen, peu d'attention est donnée à ce problème qui est étroitement lié aux changements de mode de vie et à la transition démographique. Les mesures de prévention primaires et secondaires peuvent réduire les complications à court et à long terme pour les mères et leur enfants.

Les modalités de dépistage sont assez bien établies dans les pays développés, mais leur opérationnalisation dans les pays à revenu faible et intermédiaire est encore en cours d'évaluation. Des lignes directrices consensuelles formulées par la fédération internationales des gynécologues et obstétriciens (FIGO) ont été publiées fin 2015 avec des recommandations particulières pour les pays à faible revenu. Elles devraient améliorer la situation actuelle qui est toujours dominée par le manque de standardisation dans la pratique.

Au Maroc le dépistage n'est pas encore effectué de façon systématique malgré le fait que les services de santé soient disponibles pour la prise en charge de ce problème de santé. Dans la première phase de ce projet, menée de juillet à décembre 2015, nous avons fait un état des lieux de la situation actuelle du dépistage et de la prise en charge du DG dans les structures sanitaires sélectionnées dans deux provinces, Marrakech et El-Haouz. En utilisant une méthode mixte, nous avons exploré les conditions et le degré de dépistage pendant la CPN ainsi que les connaissances et la pratique des prestataires en ce qui concerne le dépistage et la prise en charge du diabète gestationnel ainsi que les connaissances des femmes enceintes. Les résultats mettent en évidence différents défis, lesquels sont un obstacle à un dépistage universel : le manque de connaissances, l'indisponibilité des guides, des délais au niveau des laboratoires, le manque de communication et de collaboration entre les différents niveaux et entre les différents prestataires de soins de santé et l'ignorance de la femme à l'égard de la raison d'être et l'importance du dépistage du DG.

Afin d'améliorer la situation actuelle de la détection et de la PEC du DG au Maroc, en collaboration avec le groupe de recherche sur le diabète gestationnel au Maroc (comprenant les représentants du ministère de la Santé, les organismes de recherche, les représentants des organisations professionnelles, les médecins et les spécialistes ainsi que les acteurs de la communauté), nous allons mener une recherche d'implantation. Cette recherche évaluera les conditions de mise en œuvre d'une intervention de dépistage et de prise en charge décidée par le ministère de la santé et qui se base sur les lignes directrices de consensus de la FIGO les plus récentes, adaptées selon les résultats de notre analyse de la situation sur le DG. Cette étude a donc pour objectif d'évaluer la faisabilité d'un dépistage universel avec l'initiation du traitement initial du diabète gestationnel au niveau des centres de santé et d'analyser le coût/efficacité et les conditions pour intégrer ce modèle avec succès dans le système de soins de santé primaires existant.

Cette étude va contribuer à donner une information suffisante aux décideurs pour modifier la stratégie nationale de dépistage et de PEC de toutes les femmes enceintes affectées d'un DG et ainsi réduire le fardeau de la morbidité maternelle et infantile. Ce faisant, le Maroc préviendra la survenue de diabète de type 2, conséquence à moyen terme d'un diabète gestationnel non ou mal pris en charge.

Contexte

Le diabète gestationnel (DG) est défini par l'OMS comme une « hyperglycémie apparue ou décelée pour la première fois pendant la grossesse » (OMS, 2013). Au niveau mondial, la prévalence du DG est de 16,9% (FID, 2013). Le DG est associé à des complications survenant essentiellement dans la période périnatale. Ces complications peuvent être graves chez la mère et son nouveau-né. Une femme présentant un DG a un risque accru de 10% d'accoucher avec césarienne, de 70% de développer une hypertension artérielle et de 30 à 80% de présenter une pré-éclampsie durant la grossesse (Fadl, 2010; HAPO, 2008).

La macrosomie est la principale conséquence néonatale d'un DG. L'étude HAPO (Hyperglycaemia and Adverse Pregnancy Outcomes), effectuée sur 25.000 femmes enceintes, a montré qu'entre 5,3 et 26,3% de femmes présentant un DG a accouché d'un nouveau-né macrosome (HAPO, 2008). Une étude menée au Maroc a montré que 31,6% des femmes diagnostiquées avec un diabète pendant la grossesse avaient un bébé macrosome (El Amrani, 2012). La macrosomie peut être responsable d'une dystocie de l'épaule, de fracture osseuse ou de paralysie du plexus brachial du nouveau-né. Le risque d'accoucher prématurément est également accru de 50-70% (Fadl, 2010 ; HAPO, 2008). D'autres complications immédiates peuvent survenir chez le nouveau-né notamment l'asphyxie, la détresse respiratoire et l'hypoglycémie en post-partum. Le diabète pendant la grossesse a également été décrit comme un facteur de risque de mortinatalité (Syed et al. 2011).

De surcroît le DG augmente de 35-50% le risque de développer une hyperglycémie au cours de grossesses ultérieures et les femmes avec DG sont sept fois plus susceptibles de développer un diabète permanent plus tard dans leur vie (Bellamy, 2009). Une étude allemande a montré que 5,5% des femmes ayant eu un DG sont diagnostiquées avec un diabète trois mois après l'accouchement (Schäfer-Graf et al. 2009). Environ 10 ans après, 14 à 40% des femmes sont devenues diabétiques (Albareda, 2003; Feig, 2008; Lauenborg, 2004), 50% le sont après 15 ans (Ryan, 2001) et 73% après 25 ans (O'Sullivan, 1989).

A long terme, le DG peut entraîner de nombreuses anomalies métaboliques (Ballas et al. 2012) avec des implications majeures pour la santé publique, en particulier pour les systèmes de santé à ressources limitées.

Enfin, les enfants nés de mères ayant présenté un DG ont un risque plus élevé de développer une obésité et un diabète (Yogev et Visser, 2009). Dans une étude menée aux Etats-Unis, le risque pour les enfants nés macrosomes d'avoir un syndrome métabolique (obésité, dyslipidémie, hyperglycémie, hypertension) à l'âge de 11 ans était doublé (Boney et al. 2005).

Au Maroc, classé comme un pays à revenu moyen inférieur avec une population totale de 33.8 millions, plus de 1,67 millions de personnes souffrent de diabète (FID 2015). La prévalence du diabète au Maroc est 7.7% (FID 2015) et on estime que le nombre devrait doubler dans les 20 prochaines années. Bien que le diabète est plus fréquent dans les zones urbaines, 34,6% des cas de diabète dans la région MENA sont signalés dans les milieux ruraux (FID 2013).

On estime que 3,4 millions de femmes en âge de procréer dans la région MENA souffrent de diabète gestationnel ou d'un diabète préexistant (FID 2013). Dans une étude réalisée entre 2008-2009 dans le CHU de Rabat, la prévalence d'un diabète gestationnel était de 8,2% (Bouhsain et al. 2014), or les chiffres au niveau national sont inconnus. Les effets des maladies non transmissibles sur la santé maternelle et néonatale n'ont pas encore été largement étudiés au Maroc, un pays qui a bien progressé vers les cibles des OMD. Avec une baisse du ratio de mortalité maternelle de 300 en 1990 à 112 pour 100 000 naissances vivantes en 2011 et un taux de fécondité de maintenant 2,2 (HCP 2014), l'objectif d'amélioration de la santé maternelle va de plus en plus porter sur la réduction de la morbidité liée à la grossesse, à l'accouchement et au post-partum.

Des recommandations de bonnes pratique ainsi que des arbres décisionnels existent et comprennent la détection du diabète gestationnel, mais les messages dans les différents guides ne sont pas uniformes. Le plan d'action marocain pour la réduction de la mortalité maternelle et néonatale recommande de mesurer la glycémie à jeun dans les centres de santé et de faire le dépistage du diabète dans les hôpitaux de référence avec les analyses de glycémie à jeun faisant partie d'un bilan gratuit (Ministère de la Santé 2011).

Lors d'un atelier tenu à l'ENSP en octobre 2014 avec différents partenaires qui travaillent dans le domaine du diabète et de la santé maternelle au Maroc, ce groupe de travail a constaté que les connaissances sur le DG au Maroc étaient très limitées et qu'il fallait tout d'abord combler le manque de connaissances concernant la détection et la prise en charge du DG au Maroc. Nous avons mené une étude transversale exploratoire utilisant une méthode mixte avec les objectifs suivants: décrire la situation actuelle du dépistage et de la prise en charge du diabète pendant la grossesse (diabète gestationnel et diabète préexistant) dans la région de Marrakech-El Haouz; identifier les problèmes liés au dépistage et à la prise en charge du DG; décrire les connaissances et comprendre les perceptions du diabète gestationnel chez les femmes enceintes et chez les professionnels de la santé et explorer l'information reçue par les femmes sur le DG pendant la CPN. Les données ont été recueillies dans 15 centres de santé, les hôpitaux de référence (provincial, régional et le CHU), et dans trois cabinets privés.

Les résultats de l'analyse de situation indiquent que le dépistage est déficient au Maroc. Bien que la glycémie à jeun fasse partie de la détection au niveau de la CPN, les glycémies dans le secteur public se font uniquement dans les laboratoires hospitaliers sur rendez-vous et selon une règle de quota par jour. Cela signifie que les femmes doivent parfois attendre 2-4 semaines pour obtenir un rendez-vous pour le bilan qui comprend la glycémie à jeun. Les tests de tolérance au glucose par voie orale ne sont pas faits souvent, sauf sur demande spécifique. Si les femmes sont diagnostiquées avec une hyperglycémie, elles sont envoyées chez un gynécologue et / ou chez un endocrinologue. Obtenir des rendez-vous avec les endocrinologues du secteur public peut prendre un certain temps car ils sont très occupés. Cependant, les femmes vont alors faire leurs tests dans le secteur privé. Jusqu'à présent, toutes les femmes atteintes de diabète gestationnel qui ont besoin de médicaments sont traitées avec l'insuline, malgré les recommandations internationales indiquant que l'antidiabétique oral, la metformine, est possible comme traitement en première ligne. Considérant que le temps d'intervenir sur le plan thérapeutique durant la grossesse est limité à seulement quelques mois, tout retard dans la détection et la PEC du DG entrave une prise en charge à temps qui permettrait de réduire les complications liées au DG que ce soit chez la mère ou chez le nouveau-né.

Projet de recherche proposé

Les directives nationales sur la détection et la PEC du DG sont en train d'être révisées en fonction des dernières recommandations internationales afin de réaliser un modèle adapté pour le dépistage et la PEC du diabète gestationnel qui peut être intégré dans la CPN au niveau des structures de santé primaires. Notre projet évaluera les conditions de mise en œuvre d'une stratégie de dépistage et de prise en charge du DG décidée par le ministère de la santé et qui se base sur les lignes directrices de consensus de la FIGO les plus récentes, et adaptées selon les résultats de notre analyse de la situation sur le DG. Cette étude a donc comme pour objectif d'évaluer la faisabilité d'un dépistage universel avec l'initiation du traitement initial du diabète gestationnel au niveau des centres de santé et d'analyser le coût/efficacité et les conditions pour intégrer ce modèle avec succès dans le système de soins de santé primaires existant.
L'étude repose sur deux volets principaux :

- A. Le renforcement des capacités des prestataires de soins de santé primaires dans le dépistage et la prise en charge initiale des femmes affectées par un DG

- B. Une recherche efficacité-implantation pour évaluer ce modèle de dépistage et prise en charge du DG décentralisé et l'évaluer dans un échantillon de structures de santé de soins primaires en utilisant un design d'essai randomisé contrôlé par grappes.

Objectifs et hypothèses

Objectif général

Cette étude vise à évaluer l'effet et les conditions d'implantation d'une stratégie adaptée au contexte marocain pour la détection du diabète gestationnel et de sa surveillance dans les structures de soins de base afin d'améliorer l'accès des femmes enceintes au dépistage et ainsi contribuer à une réduction de la morbidité maternelle et néonatale au Maroc.

Hypothèses

La détection et la prise en charge du diabète gestationnel sont fragmentées et éparpillées entre des différents niveaux de santé. Ceci représente un obstacle majeur pour une détection à temps du diabète gestationnel et rend difficile le suivi des patientes atteintes de DG.

La détection du diabète gestationnel chez toutes les femmes enceintes (détection universelle) et la prise en charge des patientes avec DG non-compliquées au niveau des soins primaires va:

- Augmenter le nombre des femmes détectées avec un DG;
- Faciliter la prise en charge plus précoce des femmes avec DG;
- Améliorer les connaissances des prestataires de soins et des femmes sur le DG et assurer la conformité au traitement recommandé des femmes affectées par un DG;
- Réduire les complications du DG chez le nouveau-né et le nombre de macrosomes ;
- Réduire l'incidence du diabète (mères et enfants) à long terme.

Objectifs spécifiques

1) Améliorer l'accès universel à la détection du DG et sa surveillance;
2) Renforcer les compétences des prestataires des structures de soins de santé primaires concernant la détection, la prise en charge initiale et le suivi des patientes avec DG;
3) Mesurer la prévalence du diabète gestationnel dans les populations desservies par les structures de santé investiguées;
4) Explorer les effets de la prise en charge (PEC) décentralisée du DG sur le poids/la glycémie pendant la grossesse et sur la proportion de complications autour de l'accouchement;
5) Investiguer les effets de la prise en charge (PEC) décentralisée du DG sur le poids et l'état du nouveau-né à sa naissance (Apgar);
6) Examiner les effets de la PEC décentralisée sur les changements de mode de vie des femmes avec DG;
7) Comparer les perceptions des femmes suivies selon les différents protocoles quant à leur traitement et la qualité des soins reçus;
8) Mesurer la proportion des femmes re-testées à 6 semaines en post-partum pour un diabète;
9) Evaluer l'adhésion aux lignes directrices dans les structures d'intervention
10) Evaluer l'acceptabilité du dépistage et du suivi au niveau des structures de soins primaires par les clientes et les prestataires et comprendre les facteurs facilitateurs et les défis pour la pratique quotidienne;

11) Evaluer les coûts de l'intervention pour le système de santé et pour les femmes enceintes;

12) Générer l'évidence sur les meilleures pratiques et les défis rencontrés.

Méthodes

Devis de la recherche

Nous allons mener une recherche d'implantation afin de tester la mise en oeuvre d'une nouvelle stratégie par un essai randomisé contrôlé par grappes. Ce type de recherche a le double objectif d'évaluer l'efficacité clinique et en même temps la mise en œuvre d'une nouvelle stratégie en collectant information sur le dépistage et la PEC des femmes avec DG au niveau des centres de santé (Curran et al., 2012).

Durée

L'étude commencera après approbation par les comités d'éthique (CERB, Rabat et IRB (IMT) et UZA Anvers). La recherche se déroulera sur 12 mois.

Sites

L'étude se déroulera dans la préfecture de Marrakech et la province d'Al-Haouz (Figure 1). La région de Marrakech Safi est la deuxième région du Maroc en matière de nombre de décès identifiés par le système de surveillance des décès maternels et l'une des régions prioritaires du ministère de la santé (MS 2013). La préfecture de Marrakech, une préfecture essentiellement urbaine est assez représentative de toute situation urbaine. La province d'El Haouz, une province rurale, montagneuse et où l'accès aux soins est difficile rassemble toutes les difficultés rencontrées dans les provinces rurales au Maroc. La région d'Al Haouz est par ailleurs bien connue par des chercheurs qui ont documenté la morbidité pré- et post-partum.

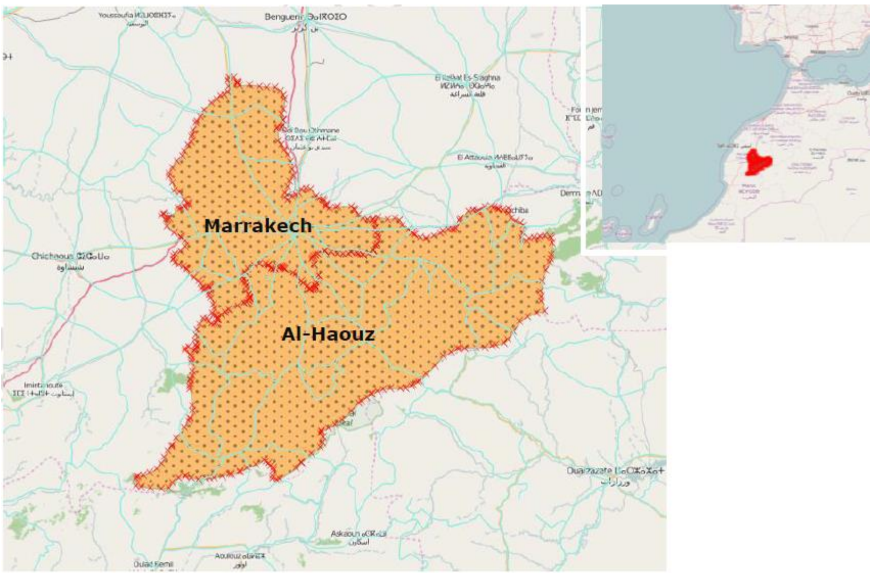

Figure 1: Préfecture de Marrakech (urbain) et province d'Al Haouz rural

Nous allons mener la recherche à Marrakech et Al Haouz dans 20 structures de soins primaires sélectionnées selon le critère du nombre de consultations prénatales égal ou supérieur à 30 consultations (nouveaux cas) par mois. La sélection des structures sera réalisée de façon aléatoire simple (Tableau 1). Nous allons inclure 10 structures (5 à Marrakech et 5 à Al Haouz) dans le groupe intervention, dans lequel le nouveau modèle de détection et prise en charge va être mis en œuvre. Dix structures (5 à Marrakech et 5 à Al Haouz) vont servir de témoins.

Tableau 1: Structures des soins de santé primaires à Marrakech et Al Haouz avec 30 ou plus CPN/mois en moyenne (SIAAP, 2014).

**Al Haouz**				**Marrakech**			
Structures SANITAIRE	*Type structure*	*NC CPN /an 2014*	*NC CPN /mois moyenne*	*Structures SANITAIRE*	*Type structure*	*NC CPN /an 2014*	*NC CPN /mois moyenne*
**OURIKA**	CSRA	566	47	**SID ZOUINE**	CSCA	559	47
**ASNI**	CSRA	692	58	**OUDAYA***	CSCA	735	61
**TAMESLOHT***	CSRA	774	64	**KETTARA***	CSCA	358	30
**TOUAMA**	CSRA	504	42	**ELMASSIRA***	CSUA	728	61
**TAKERKOUST**	CSRA	663	55	**Y. BEN TACHFINE***	CSUA	653	54
**AIT OURIR***	CSUA	659	55	**MHAMID***	CSU	807	67
**AMIZMIZ***	CSUA	1190	99	**MAATALAH**	CSU	406	34
**TAZART***	CSC	508	42	**AKIODE**	CSU	404	34
**AIT AADEL**	CSC	556	55	**KOBBA**	CSU	597	50
**TAHANOUT***	CSU	621	51	**OASIS**	CSU	458	38
				**ECHOUHADA**	CSU	407	34
				**AIN ITTI**	CSU	470	39
				**DAR TOUNSI**	CSU	408	34
				**TAMNSOURT**	CSU	454	38
				**SAADA***	CSC	410	34
				**HARBIL**	CSC	359	30
				**C/S 44**	CSC	551	46
				**O-S-B**	CSC	557	46

CSRA: Centre de santé rural avec maison d'accouchement ; CSUA: Centre de santé urbain avec maison d'accouchement ; CSCA: Centre de santé communautaire avec maison d'accouchement; CSR: Centre de santé rural; CSU: Centre de santé urbain; CSC: Centre de santé communautaire; *structures incluses dans l'étude analyse de la situation 7-12.2015.

Population

Nous avons l'intention de dépister pour DG toutes les femmes enceintes qui viennent pour la CPN dans les 20 structures incluses dans cette recherche. Toutes les femmes diagnostiquées avec un diabète gestationnel seront recrutées sur une période de 8 à 12 semaines dans chaque groupe. Le suivi des patientes sera assuré jusqu'à 6 semaines après l'accouchement de la dernière femme recrutée dans l'échantillon.

Les critères d'inclusion sont les suivants :

- Toutes les femmes enceintes qui se présentent à la CPN dans les structures incluses (dépistage);
- Femmes diagnostiquées avec un diabète gestationnel dans les structures incluses (suivi);
- Consentement éclairé pour participer à cette étude.

Les critères d'exclusion sont les suivants:

- Femme déjà connue diabétique de type 1 ou 2
- Femmes qui n'ont pas donné leur consentement de participer à cette étude.

Stratégie d'échantillonnage

Dans la littérature internationale (Langer at al. 2005, Landon et al. 2009) une différence de poids à la naissance entre les femmes avec DG non-traitées versus celles traitées est mise en évidence. Langer (Langer et al., 2005) ont pu montrer dans une étude cas-témoin incluant 1100 cas et 555 témoins et qui a été menée en 1990 et 1999 aux Etats-Unis, qu'il y avait un différence de poids à la naissance de 330 grammes entre les nouveau-nés des mères non traitées par rapport aux enfants des femmes traitées pour DG. Pour le calcul de notre échantillon nous supposons qu'un traitement selon les nouveaux protocoles immédiatement initiés par les prestataires au niveau du CS engendra une différence de poids des nouveau-nés qui va être inférieur par rapport au poids des nouveau-nés des femmes prises en charge de manière habituelle (témoins).

Prenant en compte l'effet cluster en calculant la taille de notre échantillon (test bilatéral d'une comparaison des moyennes) nous supposons que le poids des nouveau-nés dans la groupe «témoins » est de 3700g (ET +/-500g) et que le poids dans le groupe « intervention » est de 3400g (ET+/- 500g). Ajusté pour un design cluster avec 20 structures incluses), un pouvoir de 80% et alpha de 0,05 et un coefficient intra-classe

(rho) de 0,1 nous arrivons à un échantillon de 8 cas de DG par centre [20 centres inclus], donc dans chaque groupe (intervention et témoins) nous avons besoin de 75 femmes diagnostiquées avec un DG.

En se basant sur les chiffres des centres de santé de l'année 2014 (SIAAP, 2014), avec un nombre moyen de 48 consultations prénatales dans les structures ayant 30 CPN par mois ou plus, nous arriverons à environ 2000 nouvelles inscriptions par mois à la CPN dans les 20 structures. Si on assume une prévalence de 8,2% (Bouhsain et al., 2014) nous devrons au moins inclure 900 femmes enceintes dans chaque groupe pour détecter un diabète gestationnel chez 75 femmes par groupe.

Déroulement des différents volets

Volet A : Renforcement des capacités des prestataires de soins de santé dans le dépistage et la gestion initiale des femmes affectées par un DG

Après validation du protocole modifié sur la détection et la PEC du DG et du guide marocain sur la nutrition des femmes avec DG par le comité scientifique, nous allons former les prestataires dans les structures de santé sélectionnées dans son application pratique. Un plan du contenu de la formation est montré dans le tableau 2.

Tableau 2: Plan de formation

Formation	Cadres invités	Contenue
**Jour 1**	Infirmières SM, sages-femmes, médecins généralistes des structures (groupe intervention)	○ Informations générales sur le DG, le fardeau de morbidité et conséquences pour mère et nouveau-né<br>○ Information et conseil sur étude DG<br>○ Comment mesurer la glucose capillaire<br>○ Comment et quand effectuer le HGPO 75g<br>○ Diagnostic DG<br>○ Comment calibrer un glucomètre<br>○ Nutrition: Calcul des besoins caloriques et planification d'un régime alimentaire<br>○ Informations sur régime avec la nourriture localement disponible<br>○ Counseling sur l'alimentation et l'exercice<br>○ Counseling surveillance de la glycémie<br>○ Counseling sur documentation des mouvements fœtaux par les femmes<br>○ Re-tester en post-partum des mères atteintes de diabète gestationnel<br>○ Documentation
**Jour 2**	Médecins généralistes des structures (groupe intervention)	○ Prise en charge médicale: quand commencer le traitement<br>○ Surveillance glycémie<br>○ Intervalle et contenu de suivi<br>○ Suivi croissance fœtale<br>○ Comment aligner suivi gynécologique de routine avec suivi de DG<br>○ Comment administrer la metformine/insuline<br>○ Quand consulter endocrinologue<br>○ Fiche de référence et communication<br>○ Reconnaissance et traitement d'une hypoglycémie (mère et nouveau-né)<br>○ Assurer suivi en post-partum et au long du cycle de la vie<br>○ Avis pré-conceptionnel des femmes affectées

Volet B : Recherche efficacité-implantation pour piloter ce nouveau modèle et l'évaluer dans un échantillon des structures des soins de santé primaires dans les deux sites de Marrakech et Al-Haouz.

Nous allons mener un essai randomisé contrôlé par grappes avec une groupe intervention et un group témoins. Dans le groupe témoins (10 structures- 5 à Marrakech et 5 à Al-Haouz), les femmes vont être dépistées et prises en charge comme d'habitude, ce qui inclut généralement un bilan dans un laboratoire et la prise en charge de cette femme par un spécialiste.

Dans les structures d'intervention (10 structures – 5 à Marrakech et 5 à Al-Haouz) nous allons piloter le nouveau modèle du dépistage et la prise en charge initiale du DG au niveau des structures des soins de santé primaires. Un test de glycémie capillaire à jeun sera effectué à la première visite prénatale déjà au niveau du centre de santé. Les femmes vont être réinvitées à un âge de grossesse entre 24 et 28 semaines pour un test d'hyperglycémie provoquée (HGPO) avec 75g de glucose qui sera fait dans les centres de santé.

Le test de glycémie par voie capillaire est disponible dans les centres de santé et fait aussi partie du bilan standard pour les femmes enceintes au niveau des laboratoires par voie veineuse. Le HGPO, par contre, est actuellement effectué seulement sur commande spécifique dans certains laboratoires. Pour cette étude pilote nous n'allons donc pas introduire des nouveaux tests mais nous allons décentraliser ceux qui sont déjà recommandés dans les directives nationales (MS, ANAM 2013) et les rendre plus accessibles pour toutes les femmes enceintes déjà au niveau des centres de santé (voir arbre décisionnel figure 2- à titre d'exemple avant validation)

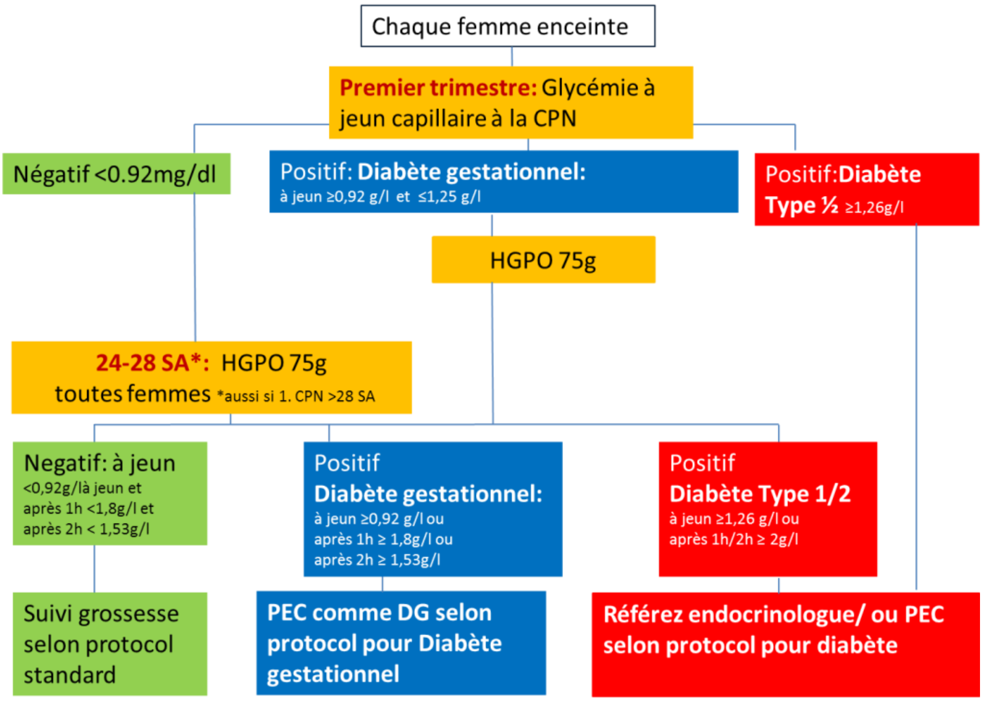

Figure 2: Exemple d'arbre décisionnel proposé pour la détection universelle du DG Modifed according to Hod et al (2015) and Indian Ministry of Health and Family Welfare (MOHFW 2014)

Dans les structures d'intervention, toutes les femmes enceintes vont être informées pendant leur CPN de l'importance d'un dépistage du diabète gestationnel. Celles diagnostiquées avec un DG seront informées sur la prise en charge selon le nouveau protocole établi. Nous demandera le consentement éclairé des femmes enceintes pour être incluses dans cette étude et pour être contactées par nos enquêtrices et enquêteurs pendant la grossesse et après l'accouchement. Toutes les femmes dépistées avec DG recevront des conseils sur un régime alimentaire adapté aux conditions locales et sur l'exercice physique modéré pendant la grossesse ; la PEC sera effectuée selon le protocole établi dans l'étude (voir figure 3- exemple d'arbre) après sa validation par le comité scientifique ce qui va également définir les intervalles pour le suivi des femmes atteintes par un DG au niveau de leur centre de santé. La surveillance obstétricale de routine sera assurée par le gynécologue comme d'habitude. Les femmes avec DG seront invitées à être re-testées pour un diabète six semaines après leur accouchement (programmé lors de leur visite de vaccination de leur enfant au centre de santé).

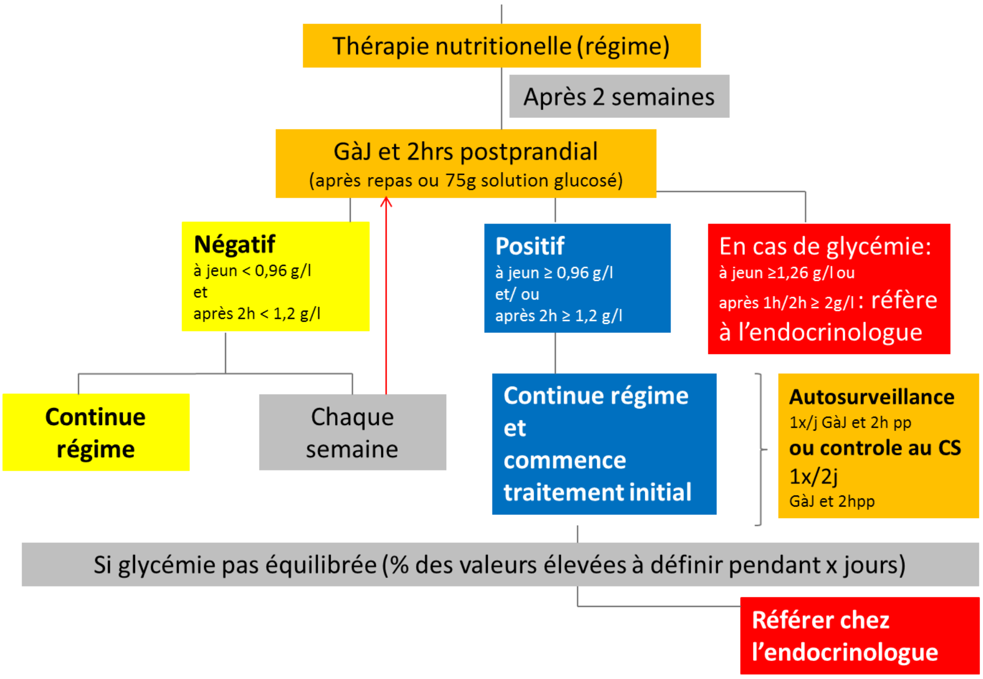

Figure 3: Exemple d'arbre décisionnel proposé pour la PEC des femmes avec DG au niveau du CS. Modifed according to Hod et al (2015) and Indian Ministry of Health and Family Welfare (MOHFW 2014)

Evaluation

Pour évaluer l'introduction des protocoles de détection et de prise en charge du DG aux centres de santé nous allons collecter les données en relation avec les objectifs suivants :

1. Améliorer l'accès universel à la détection du DG et sa surveillance.
<u>Indicateurs :</u> (Voir tableau 3)

Tableau 3: Sélection des indicateurs préliminaires

a.	*No. de femmes testées pour DG avec glycémie à jeun/ No. femmes à la CPN (nouvelles inscriptions).*	*k.*	*No. de femmes avec DG sous régime seulement/ No. des femmes avec DG suivies*
b.	*Âge gestationnel (moyen) des femmes testées avec GàJ*	*l.*	*d. Âge gestationnel (moyen) des femmes testées avec HGPO*
c.	*No de femmes testées pour DG avec HGPO 75g / No. des femmes à la CPN*	*m.*	*No. de femmes avec DG sous traitement médical/No. des femmes avec DG suivies*
d.	*No. de femmes diagnostiquées avec une hyperglycémie (DG/DM)/ No. des femmes testées*	*n.*	*Intervalle (moyen; en semaines) entre diagnostic et commencement traitement nutritionnel*
e.	*Âge gestationnel (moyen) des femmes diagnostiquées avec une hyperglycémie*	*o.*	*Âge gestationnel (moyen) au commencement du traitement*
f.	*No. de femmes mises sous régime*	*p.*	*Intervalle entre commencement régime et commencement traitement médical*
g.	*No. de femmes référées chez un endocrinologue/no. des femmes diagnostiquées avec DG*	*q.*	*No. de femmes avec DG suivies perdues de vue/ No. des femmes avec DG suivies*
h.	*No. de femmes avec DG suivies au niveau du CS/ No. des femmes diagnostiquées avec DG*	*r.*	*Intervalle (moyen; en semaines) entre diagnostic et accouchement*
i.	*No. de suivis par femme avec DG au niveau du CS/ No. des femmes diagnostiquées avec DG au niveau de CS*	*s.*	*Intervalle (moyen; en semaines) entre commencent traitement (nutritionnel) et accouchement*
j.	*Intervalle (moyen; en semaines) suivi*	*t.*	*Intervalle (moyen; en semaines) entre commencent traitement (médicamenteux) et accouchement*

2. Renforcer les compétences des prestataires des structures des soins de santé primaires dans la détection, la prise en charge initiale et le suivi des patientes avec DG.

Indicateurs :
Évaluation des connaissances-pratiques sur la détection et la PEC du DG avant et après la formation (pré et post-test immédiat et 4 semaines après la formation).

3. Calculer la prévalence du diabète gestationnel.

Indicateur:
No. des femmes testées positives pour un DG/ No. des femmes testées pour DG.

4.Investiguer les effets de la prise en charge (PEC) du DG au niveau du centre de santé sur le poids/la glycémie de la femme pendant la grossesse et sur la proportion de complications autour de l'accouchement

Indicateurs:
Niveau moyen de glycémie pendant le suivi ;
Poids au début de la grossesse (IMC) ;
Evolution du poids pendant la grossesse (catégories IMC) ;
Voie d'accouchement ;
Complication pendant l'accouchement : dystocie épaules, pré-éclampsie, travail prolongé, mort-né, accouchement prématuré, macrosomie.

5. Investiguer les effets de la prise en charge (PEC) du DG en comparant les deux groupes (groupe intervention et témoins) sur le poids et l'état du nouveau-né à la naissance.

Indicateur:
Poids du nouveau-né à la naissance ;
APGAR du nouveau-né à sa naissance (1' et 5').
Morbidité associée (hypoglycémie, détresse respiratoire, cardiomyopathie..)

6. Evaluer les effets de la PEC décentralisée sur les changements de mode de vie des femmes avec DG

Indicateur:

Degré d'accord ou désaccord vis-à-vis d'une affirmation concernant les modes de vie (changement de régime ; adhérence à une pratique d'exercice physique) en utilisant des échelles de Likert (Questionnaire adressé aux femmes avec DG qui ont accouché)

7. Analyser les perceptions des femmes suivies selon les différents protocoles sur le traitement et la qualité des soins reçus

Indicateur :

Degré d'accord ou désaccord vis-à-vis d'une affirmation en utilisant des échelles de Likert dans un questionnaire adressé aux femmes avec DG qui ont accouché

8. Mesurer la proportion des femmes re-testées pour un diabète en post-partum tardif (6 semaines en post-partum);

Indicateur :
No. femmes avec DG re-testées post-partum tardifs (jusqu'à 6 semaines)/no. des femmes diagnostiquées avec DG.

9. Evaluer l'adhésion aux lignes directrices concernant la détection du DG dans les structures d'intervention

Indicateur:
Audit de la pratique de détection du DG

10a. Evaluer l'acceptabilité du dépistage du DG et du suivi au niveau des structures des primaires par les femmes enceintes et comprendre les facteurs facilitateurs et les défis pour la pratique quotidienne ;

Entretiens semi-structurés avec 30 femmes diagnostiquées avec DG au niveau des structures incluses (15 interventions et 15 témoins- chaque cinquième femme incluse dans chaque groupe)

10b. Evaluer l'acceptabilité du dépistage du DG et le suivi au niveau de soins de santé primaire par les prestataires de santé et comprendre les facilitateurs et les défis pour leur

pratique quotidienne et évaluer l'adhésion des soignants et la facilité de nouveau protocole;

Focus groupes avec les prestataires de soins qui sont chargés de la détection et du suivi des femmes avec DG dans leur structure de soins de santé primaires (structures 'intervention').

11) Evaluer les coûts de l'intervention pour le système de santé et pour les femmes enceintes:

<u>Indicateurs:</u>
Calcul des coûts de la décentralisation de la prise en charge du DG.
Pour le service de santé :

- *Coûts additionels et coûts de l'application actuelle de la stratégie*
- *Amortissement des coûts de l'équipement ;*
- *coûts récurrents: tests de laboratoire, dépenses pour médicaments, temps passé pour le dépistage et la prise en charge*

Pour les patientes :

- *des coûts directs pour les femmes, y compris voyage, tests de laboratoire, les dépenses pour médicaments et matériel*

(Source : Coûts de l'intervention, Questionnaire structuré et entretiens avec femmes atteintes du DG).

Collecte de données et outils

Après la formation des prestataires, les femmes enceintes dans les différentes structures vont être dépistées pour DG selon le protocole (groupe intervention : nouveau protocole; groupe témoins: protocole/pratique habituelle). Les femmes qui sont testées positives pour DG vont être suivies selon les protocoles (groupe intervention : nouveau protocole ; groupe témoins : protocole/pratique habituelle). Après avoir obtenu le consentement des femmes, les coordonnées de chaque femme diagnostiquée avec DG vont être documentées par les agents de santé pour que les enquêteurs puissent les contacter plus tard.

Les données quantitatives

La collecte de données quantitatives au niveau des CS/CS-MA sera effectuée par des enquêteurs qui vont visiter la structure mensuellement et qui vont rester en contact téléphonique avec le personnel impliqué dans la détection et le suivi des femmes.

Les données seront extraites grâce à une fiche d'extraction de données et entrées dans une base de données format Excel mensuellement. L'investigateur principal va être en contact avec l'équipe du terrain et superviser la collecte des données.

Les données collectées au niveau de la structure incluent les informations mensuelles sur le nombre des femmes dépistés et suivi et le matériel utilisé. Pour collecter des informations individuelles sur les femmes dépistées/suivies nous allons mettre une fiche des données spécifiquement sur le DG dans le carnet de santé de chaque femme incluse

dans cette étude pour que l'agent de santé mette les informations additionnelles vis-à-vis du DG sur cette fiche. Par une enquête après l'accouchement, les perceptions des femmes sur la qualité des soins, les implications du DG sur leur mode de vie mais aussi les dépenses vont être évaluées. Des enquêtes vont être conduites par des enquêteurs 6 à 8 semaines post-partum soit au domicile de la femme, dans un endroit choisi par la femme à un moment approprié, ou soit par téléphone si une visite n' est pas faisable.

Les données concernant l'évaluation des connaissances-pratiques sur la détection et la PEC du DG avant et après la formation (pré et post-test) vont être collectées à l'aide d'un questionnaire structuré distribué parmi les participants de la formation avant et après la formation.

Les données qualitatives

Pour les données qualitatives, les stratégies de collecte de données et les outils mobilisés sont les entretiens semi-directifs avec un échantillon des femmes incluses dans cette étude (chaque cinquième femme) afin d'évaluer leur perception du déroulement de la détection du DG au niveau de leur structure, leur perception du suivi, la qualité des soins reçus, les modifications de la qualité de vie après le diagnostic et les coûts impliqués (après accouchement). Nous allons aussi mener deux focus-groupes (FG) avec les prestataires chargés du dépistage du DG et du suivi des femmes avec DG dans les structures d'intervention pour mieux connaître leurs perceptions et leur expérience de ce nouveau modèle, ses avantages et ses défis pour leur pratique quotidienne (trois à six mois après le début de l'intervention). Un FG va avoir lieu à la direction régionale de la santé à Marrakech et un FG à la direction provinciale d'Al Haouz.

En outre nous allons interviewer 10 informateurs-clés (le nombre dépendra de la saturation de l'information reçue) pour avoir plus d'information sur leur avis concernant ce nouveau modèle et le potentiel pour le mettre à échelle.

Nous allons faire des observations pendant une journée de CPN dans les structures incluses dans l'étude (5 CPN par centre) pour vérifier le temps passé pour le dépistage et la prise en charge (utilisé pour le calcul des coûts). Dans les structures d'intervention nous allons en même temps évaluer l'adhésion aux lignes directrices concernant le dépistage du DG chez les femmes enceintes

Toutes les entretiens et focus groupes vont être menés à un moment appropriés pour les participants et les frais du transport pour venir à l'entretien vont être remboursés. Les guides d'entretiens et les guides de FG sont provisoires puisque ils seront adaptés au cours du processus itératif de la méthodologie qualitative.

Les entretiens avec les prestataires seront menés en Français, et avec les femmes en Arabe/ Berbère par un enquêteur formé. Ils seront enregistrés sur support numérique après avoir demandé l'approbation des participants.

Tableau 4: Données à collecter

**TEMOINS**		**INTERVENTION**	
5 CS/CSMA urbain	5 CS/CSMA rurale	5 CS/CSMA urbain	5 CS/CSMA rurale
		Pré- et post-test prestataires	

Données structure mensuelles	Données structure mensuelles
Données individuelles (75+)	Données individuelles (75+)
Observation consultations CPN (5 CPN par centre)	Observation consultations CPN (5 CPN par centre)
Dépistage et suivi des femmes diagnostiquées avec DG: Enquête (75+)<br>Entretiens structurés (chaque 5ème femme incluse; 15)	Dépistage et suivi des femmes diagnostiquées avec DG: Enquête (75+)<br>Entretiens structurés (chaque 5ème femme incluse; 15)
	Focus groupes avec prestataires (2; 1 Marrakech, 1 Al Haouz)
Entretiens avec informateurs clés (locale/régional; 10)	

Analyse des données

Analyse des données qualitatives

Les entretiens et les focus groupes seront enregistrés, traduits et transcrits par la suite. Les données seront traitées avec le logiciel NVIVO10. Une analyse de contenu thématique sera réalisée à partir d'un arbre thématique a priori constitué à partir des objectifs de la recherche. Cet arbre thématique initial pourra intégrer de nouveaux thèmes émergents lors des entretiens ou des focus groupes (approche mixte : inductive et déductive).

Analyse des données quantitatives

Les données quantitatives feront l'objet d'une double-saisie et elles seront analysées avec le logiciel Stata 13IC (College Station Texas USA)/ SPSS Version 21. Des statistiques descriptives seront réalisées en fonction des échelles de mesure de chacune des données collectées.

La combinaison des données quantitatives et qualitatives et la diversité des méthodes de collecte de données permettront de trianguler les données et contribueront ainsi à renforcer la validité interne de l'étude.

Surveillance, supervision et contrôle de la qualité des données

Toutes les données quantitatives seront collectées par deux enquêtrices formées pour recueillir les données et seront supervisées pendant la durée de l'enquête par l'investigateur principal qui sera sur place. Les entretiens avec les informateurs clés et lors des focus groupes avec les prestataires vont être menés par l'investigateur principal en français assisté par l'enquêteur. Les enquêtes et les entretiens structurés avec les femmes vont être menés par des enquêtrices en arabe ou berbère. Nous espérons réduire le refus de participer à l'étude par une attitude conviviale et une explication claire de l'importance de l'étude. Le nombre de refus sera quantifié et les raisons du refus seront documentées. La qualité de saisie des données sera assurée par double entrée contrôlée.

Assurance qualité

Pour assurer la qualité du projet, un comité d'experts sera mis en place pour suivre le projet et assurer sa mise en œuvre. Le comité d'experts est impliqué dans: (1) l'adaptation des lignes directrices marocaines vers les dernières recommandations de la

FIGO; (2) le développement d'outils de formation pour les professionnels de la santé ; (3) pour surveiller le modèle spécifique au contexte des soins du diabète gestationnel. Des réunions trimestrielles régulières auront lieu entre l'équipe de projet et le groupe de travail consultatif chargé de fournir des mises à jour sur le processus. Les investigatrices principales (BU/BA) vont superviser étroitement la mise en œuvre du projet et être en communication directe avec les enquêteurs et les responsables des structures pendant la mise en œuvre du projet.

Considérations éthiques

Cette étude sera soumise à l'examen et à l'approbation formelle de l'IRB/ITM, UZA à Anvers et au comité d'éthique de l'Université de Rabat. Aucun des participants ne sera recruté et aucune activité liée à l'étude entamée avant l'obtention de l'approbation écrite de ces organismes. L'étude sera menée en respectant les principes énoncés dans la Déclaration d'Helsinki, tous les règlements applicables et selon les normes scientifiques internationales établies.

Obtention du consentement éclairé

Dans notre étude nous allons veiller à obtenir le consentement éclairé des femmes incluses dans l'étude et des interviewés au niveau des structures. Nous allons nous assurer que les participantes recrutées sont bien conscientes i) que leur participation est volontaire ; ii) qu'elles peuvent poser toutes les questions qu'elles souhaitent et qu'elles recevront des réponses compréhensibles et utiles pour décider de leur participation ou non ; iii) qu'elles peuvent se retirer à tout moment de l'étude et reprendre les données de l'étude les concernant n'importe quand et sans conséquences pour elles. Après lecture de la note d'information, l'interviewer invite les personnes qui acceptent de participer à la recherche à prendre connaissance du formulaire de consentement éclairé et à le signer ou à y laisser leur empreinte digitale (en fonction de leur niveau d'instruction). Si un répondant est incapable de lire ou d'écrire, une signature d'un témoin du consentement éclairé sera obtenue. Le témoin sera choisi par le participant.

Sécurisation des données

L'accès aux données sera restreint aux chercheurs impliqués dans l'étude. Les enquêteurs feront en sorte de protéger l'anonymat des enquêtes et la confidentialité des informations recueillies, en limitant l'accès à ces informations. En aucun cas, les enquêteurs ne pourront divulguer les informations recueillies lors de l'enquête. Les données seront gardées sous-clé et les données électroniques dans un fichier protégé par mot de passe. L'utilisation des données dans le cadre d'autres recherches sera sujette à une procédure standardisée de vérification des objectifs de la recherche et à l'accord des comités d'éthique. Nous sommes conscients que nous allons accéder à des dossiers médicaux avec des données à caractère personnel. Les enquêteurs et toutes les autres personnes qui auront accès aux données signeront un engagement sur l'honneur de respecter la confidentialité absolue et de protéger l'anonymat des participants.

Risques

Les nouveaux protocoles de dépistage du DG et de la prise en charge des femmes ayant un DG seront validés par des sociétés savantes avant d'être appliqués dans les structures.

Les tests de glycémie seront effectués dans le cadre de la prestation de routine des services de santé, selon les standards approuvés et validés par un comité scientifique au niveau national. Nous n'allons pas tester de nouveaux produits thérapeutiques ou des diagnostics qui ne font pas encore partie des pratiques du système de santé au Maroc. Dans les structures d'intervention nous allons appliquer le test de tolérance au glucose (HGPO 75g) pour le diagnostic du DG, un test recommandé selon les directives nationales (MS, ANAM 2013) et internationales sur la détection du DG (Hod et al, 2015). Ce test a comme risque des vomissements à cause de l'hyper-osmolarité de la solution glucosée, une complication qui peut affecter 3,7% des femmes (Mohan et al., 2014). Mesurer la glycémie par un prélèvement capillaire pendant la détection et le suivi peut être associé à une douleur modérée par la piqûre du doigt et accompagnée par une sensation de brûlure.

Avantages

La participation à cette étude a comme avantage que les femmes dans les structures d'intervention vont bénéficier d'un test de dépistage qui correspond aux dernières directives de consensus international et qui va être effectué au centre de santé qu'elles ont l'habitude d'utiliser et ainsi leur éviter de devoir se rendre à l'hôpital. Comme nous incluons seulement 10 centres de santé dans le groupe intervention, un nombre limité de femmes vont bénéficier du nouveau modèle de dépistage et des procédures de prise en charge au niveau du CS. Cependant, comme le ministère de la santé est impliqué dans la mise en place et l'évaluation du projet, basé sur les résultats de cette étude la mise à échelle des nouveaux protocoles au niveau national va suivre ce projet pilote pour faire bénéficier toutes les femmes enceintes à l'avenir de ce modèle adapté.

Utilisation des données de l'étude dans le cadre d'autres recherches

L'utilisation des données dans le cadre d'autres recherches sera sujette à une procédure standard de vérification des objectifs de la recherche et à l'accord du (des) comité(s) d'éthique.

Dissémination des résultats

Un rapport d'étude sera élaboré à la fin de l'analyse des données. Il sera soumis au Ministère de la Santé, à la Direction des Hôpitaux et des Soins ambulatoires et aux organisations qui financeront l'étude (OMS, UNFPA). La diffusion et la publication des résultats de l'étude seront faites par les chercheurs principaux à l'IMT et à l'ENSP. Des efforts seront déployés pour publier les résultats de l'étude dans un journal international (peer-reviewed).

Les résultats de cette étude seront alors présentés au niveau de la région de Marrakech-Safi lors d'une réunion avec les responsables concernés (Direction régionale de la Santé, délégations de la santé et représentants des structures et associations locales) en présence des chercheurs.

Calendrier

	M1	M2	M3	M4	M5	M6	M7	M8	M9	M10	M11	M12	M13	M14	M15	M16	M17	M18
Rédaction du protocole	■	■																
Finaliser nouveau protocole DG	■	■																
Développement outils de collecte des données		■	■															
Soumission protocole au comités d'éthique		■	■	■														
Développent matériel didactique pour formation			■	■	■													
Recrutement des enquêteurs					■													
Formation des enquêteurs					■													
Piloter outils						■												
Formation prestataires ; Évaluation CP (pré-posttest)						■												
Inclusion femmes avec DG dans cohortes							■	■										
Observation et audit practiques							■	■										
FGD prestataires et évaluation CP									■	■								
Entretiens informants										■	■							
Enquête et entretiens structurés avec femmes avec DG											■	■	■	■	■			
Suivi structures et collecte des données							■	■	■	■	■	■	■	■	■			
Analyse des données									■	■	■	■	■	■	■	■	■	
Dissémination des résultats																	■	■
Rapport final																		■

Budget

No.		€
**1.**	**Formation**	**2500 €**
1.1	Formation de 2 jours pour 40 prestataires	
**2.**	**Consumables**	**5500 €**
2.1	Bandelettes pr glucomètre 6000 (25 pièces à 100Dh)	2400€
2.2	Fongibles (1000 x 75g solutions glucosées, aiguilles, etc.)	2500€
2.3	Glucomètre (plasmacalibré) x 15 (à 400 Dh)	600€

**3.**	**Copies, materiel formation**	**1000 €**
**4.**	**Communication**	**1000 €**
4.1	Crédit téléphone prestataires / enquêteurs pdt 10 mois	22x 50 Dh/mois
**5.**	**Réunion dissémination**	**1800€**
**6.**	**Personnel**	**15200€**
6.1	Enquêteurs (2) (coûts transport/visites femmes incl.)	450Dh/jx13j/x9
6.2	Frais de déplacement IP pd 10 mois	3800€
6.3	Collecte de données localement 50x160	800€
6.4	Transcription	1000 €
**7.**	**Transport participants entretiens (70 participants)**	**1000€**
**8.**	**Dépenses imprévues (5%)**	**1200 €**
	**TOTAL**	**29400 €**

Références

Albareda, M., Caballero, A., Badell, G., Piquer, S., Ortiz, A., DeLeiva, A., Corocoy, R.(2003) Diabetes and abnormal glucose tolerance in women with previous gestational diabetes. Diabetes Care, 26, p. 1199-1205.

Bellamy L., Casas, J.P., Hingorani, A., Williams, D. (2009). Type 2 diabetes mellitus after gestational diabetes: a systematic review and meta-analysis Lancet; 373: 1773–79

Boney, C.M., Verma, A., Tucker, R., & Vohr, B. R. (2005). Metabolic syndrome in childhood: association with birth weight, maternal obesity, and gestational diabetes mellitus. Pediatrics, 115(3), p. e290-e296.

Bouhsain, S., El Kochri, S., Babahabib, M.A., Hafidi, M.H., Bouaiti, E., et al. (2014). Comparing two screening policies of gestational diabetes mellitus: The Mohammed V Training Military Hospital of Rabat (Morocco). *Gynecologie, obstetrique & fertilite*. pii: S1297-9589(13)00270-1. doi:10.1016/j.gyobfe.2013.09.006.

Curran, G. M., Bauer, M., Mittman, B., Pyne, J. M., & Stetler, C. (2012). Effectiveness-implementation hybrid designs: combining elements of clinical effectiveness and implementation research to enhance public health impact. Medical care, 50(3), 217.

El Amrani, F.Z. (2012) ; Diabète et grossesse. Thèse No. 38. Université Mohammed V : Faculté de Médicine et de Pharmacie : Rabat, Maroc.

Fadl, H., Oestlund, I., Magnuson, A., Hanson, U. (2010) Maternal and neonatal outcomes and time trends of gestational diabetes in Sweden from 1991 to 2003. Diabetic Medicine, 27, p. 103-107.

Feig, D.S., Zinman, B., Wang, X., Hux, J.E. (2008). Risk of development of diabetes mellitus after diagnosis of gestational diabetes. *Canadian Medical Association Journal;* **179**(3): 229-234.

FID (2015). *Diabetes Atlas Sixth Edition*. International Diabetes Federation.Available from: www.idf.org/diabetesatlas [Accessed 9.3.2015]

Hod M, Kapur A, Sacks DA, Hadar E, Agarwal M, Di Renzo GC et al. (2015). Management of hyperglycemia during pregnancy. Int J Gynaecol Obstet;131(S3):S190-200

Hyperglycemia and Adverse Pregnancy Outcome (HAPO) Study Cooperative Research Group. (2008) Hyperglycemia and Adverse Pregnancy Outcomes. New England Medical Journal, 358, p.

1991-2002.

Landon, M. B., Spong, C. Y., Thom, E., Carpenter, M. W., Ramin, S. M., Casey, B., et al. (2009). A multicenter, randomized trial of treatment for mild gestational diabetes. New England Journal of Medicine, 361(14), 1339-1348.

Langer, O., Yogev, Y., Most, O., & Xenakis, E. M. (2005). Gestational diabetes: the consequences of not treating. American journal of obstetrics and gynecology, 192(4), 989-997.

Lauenborg, J., et al. (2004). Increasing Incidence of Diabetes After Gestational Diabetes A long-term follow-up in a Danish population. *Diabetes Care;* **27**(5): 1194-1199.

Ministère de la Santé (2012). ENPSF 2011. Rabat, Morocco: Ministère de la Santé.

Ministère de la Santé. Enquête confidentielle sur les décès maternels au Maroc - 2010, Rabat: Ministère de la Santé du Royaume du Maroc, 2013. 42 pages.

MS, ANAM 2013 Recommandations de Bonnes Pratiques Médicales Affection Longue Durée ALD 6 (Selon l'Arrêté Ministériel) Diabète de type 2 (CIM 10/E11): Rabat: ANAM

MOHFW(2014). National Guidelines for Diagnosis & Management of Gestational Diabetes Mellitus, India. Ministry of Health and Family Welfare, Maternal Health Division.New Delhi: UNICEF

O'Sullivan, J. (1989) The Boston Gestational Diabetes Studies: Review and Perspectives. In: Sutherland, H., Stowers, J., Pearon, D. (eds.). Carbohydrate metabolism in pregnancy and the newborn IV. London: Springer.

Ryan, E. (2001) What is Gestational Diabetes? In: Gerstein, H.C., Haynes, R.B. (eds..) Evidence-Based Diabetes Care. Hamilton-London: BC Decker Inc.

Schaefer-Graf, U., Klavehn, S., Hartmann, R., Kleinwechter, H., Demandt, N., Sorger, M., Kjos, S., Vetter, K., Abou-Dakn, M. (2009) How do we reduce the number of missed postpartum diabetes in women with recent gestational diabetes? Diabetes Care, 32, p. 1960-1964.

SIAAP (2014). Système de routine, Marrakech-Tensift-Al Haouz, Morocco: SIAPP.

Syed, M., Javed, H. , Yakoob, M.Y., Bhutta, Z.A.et al. (2011). Effect of screening and management of diabetes during pregnancy on stillbirths. *BMC Public Health*; **11**(Suppl 3): S2.

WHO (2013). Diabetes factsheet [online]. Available at: http://www.who.int/mediacentre/factsheets/fs312/en/. [Accessed 17 September 2015].

Yogev, Y. and G. H. Visser (2009). Obesity, gestational diabetes and pregnancy outcome. *Seminars in Fetal and Neonatal Medicine*; 14 (2): 77–84
