# Supplementary material for: Detection and initial management of gestational diabetes through primary health care services in Morocco: An effectiveness-implementation trial
Source: PLoS One. 2018 Dec 28;13(12):e0209322. doi: 10.1371/journal.pone.0209322 (PMC6310282; doi:10.1371/journal.pone.0209322)
Supplement: S3 File — (PDF) [file pone.0209322.s003.pdf]

Improving detection and management of gestational diabetes at the primary level of care in Morocco

**Coordinating institution**	Ecole de Santé Publique (ENSP),<br>Rabat, Maroc
**Investigators:**	Bettina Utz<br>Bouchra Assarag
**Promoter:**	Vincent De Brouwere
**Protocol Number:**	1
**Title:**	Improving detection and management of gestational diabetes at the primary level of care in Morocco
**Version:**	1.0, Date 23.03.2016
**Coordinating institution:**	ENSP, Maroc
**Coordinating Investigator at ITM:**	Bettina Utz
**Department:**	Maternal and Reproductive Health Unit<br>Public Health Department
**Address:**	Institute of Tropical Medicine, Nationalestraat 155, 2000 Antwerp, Belgium
**Telephone/Fax:**	+32 3 247 6644/ +32 3 247 62 58
**Email:**	butz@itg.be

Statement of Compliance & Confidentiality

The information contained in this study protocol is privileged and confidential. As such, it may not be disclosed unless specific permission is given in writing by the ITM or when such disclosure is required by federal or other laws or regulations. These restrictions on disclosure will apply equally to all future information supplied which is privileged or confidential.

Once the final protocol has been issued and signed by the Investigator(s) and the authorized signatories, it cannot be informally altered. Protocol amendments have the same legal status and must pass through the mandatory steps of review and approval before being implemented.

By signing this document, the Investigator commits to carry out the study in compliance with the protocol, the applicable ethical guidelines like the Declaration of Helsinki and consistent with international scientific standards as well as all applicable regulatory requirements. The Investigator will also make every reasonable effort to complete the study within the timelines designated.

<u>Investigators:</u>

Bettina Utz Date: 23/03/2016

Bouchra Assarag:

<u>Promotors:</u>

Prof. Vincent De Brouwere

Table of contents

Statement of Compliance & Confidentiality 2
Summary 4
Backgound 5
Proposed research 6
Objectives and hypotheses 7
    General objective 7
    Hypotheses 7
    Specific objectives 7
Methods 8
    Research design 8
    Duration 8
    Study sites 8
    Population 10
    Sampling strategy 10
    Progress of the different research components 11
Evaluation 13
Data collection and tools 16
Data analysis 17
Monitoring, supervision and data quality control 18
Quality assurance 18
Ethical considerations 18
    Obtaining informed consent 18
    Data safety 19
    Risks 19
    Advantages 19
    Use of study data in other research 19
Dissemination of results 20
Activity chart 20
Budget 21
References 21

Summary

Gestational diabetes (GDM) and pre-existing diabetes are associated with a number of complications in pregnant women and their newborns, including pre-eclampsia, premature birth and stillbirth. In low- and lower middle-income countries, little attention is yet paid to this problem, which is closely linked to changes in lifestyle and demographic transition. Primary and secondary prevention can reduce short- and long-term complications for mothers and their children.

Screening modalities are already in place in developed countries, but in many low- and middle-income countries these are not yet routinely implemented. Consensus guidelines developed by the International Federation of Gynecologists and Obstetricians (FIGO) were published in October 2015 with specific recommendations for low-income countries. They may improve the current situation which is still dominated by the lack of standardization of practice.

In Morocco, screening is not yet performed systematically despite health services being available for the management of diabetes. In the first phase of this project, conducted from July to December 2015, we made an analysis of the current situation of screening and management of GDM in selected health centres in two districts, Marrakech and El Haouz. Using a mixed method design, we explored screening practices during ANC, assessed knowledge and practice of providers regarding the detection and management of gestational diabetes and explored the knowledge of pregnant women about diabetes occurring in pregnancy. Findings highlighted various challenges, which present an obstacle to universal screening: lack of knowledge, unavailability of guidelines, delays at the laboratory level, lack of communication and collaboration between different levels and between different health care providers and woman's lack of knowledge regarding reasons and for and importance of GDM screening.

In order to improve the current situation of detection and management of GDM in Morocco, we will, in collaboration with the national research group on gestational diabetes (including representatives of the Ministry of Health, research organizations, professional organizations, doctors and specialists as well as community stakeholders), conduct this implementation research. The research project will evaluate the implementation of a screening and management intervention supported by the Ministry of Health that is based on the most recent FIGO consensus guideline recommendations and adapted to the recent results of the situation analysis. The study aims to assess the feasibility of a universal screening approach coupled with treatment initiation for gestational diabetes at the health centre level and to analyse the cost- effectiveness as well as the preconditions for a successful integration of this model into the existing primary health care system. It will provide decision-makers with sufficient information to enable them to modify the national GDM screening and management strategy and thus reduce the burden of maternal and child morbidity. In the medium and long term it will contribute to the prevention of type 2 diabetes, a consequence of untreated or poorly managed gestational diabetes.

Backgound

Gestational diabetes (GDM) is defined by the WHO as "hyperglycemia that occurs or is detected for the first time during pregnancy" (WHO, 2013). At the global level, the prevalence of GDM is 16.9% (FID, 2013). GDM is associated with complications occurring mainly in the perinatal period. These complications can affect both the mother and her newborn. A woman with GDM has a 10% increased risk of giving birth with caesarean section, a 70% risk to develop high blood pressure and a 30-80% risk of a pregnancy complicated by pre-eclampsia (Fadl, 2010; HAPO, 2008).

Macrosomia is the main neonatal consequence of a GDM. The HAPO study (Hyperglycaemia and Adverse Pregnancy Outcomes), that included 25,000 pregnant women, revealed that between 5.3 and 26.3% of women with a GDM delivered a macrosome newborn (HAPO, 2008) . A study conducted in Morocco showed that 31.6% of women diagnosed with diabetes during pregnancy had a macrosome baby (El Amrani, 2012). Macrosomia may be responsible for shoulder dystocia, bone fracture, or brachial plexus paralysis of the newborn. Similarly, the risk of giving birth prematurely is increased by 50-70% (Fadl, 2010, HAPO, 2008). Other immediate complications that may occur include asphyxia, respiratory distress and postpartum hypoglycemia in the newborn. Diabetes during pregnancy has also been described as a risk factor for stillbirths (Syed et al., 2011).

In addition, GDM increases the risk of having a repeat GDM in subsequent pregnancies by 35-50%, and women with GDM are seven times more likely to develop permanent diabetes later in life (Bellamy, 2009). A study conducted in Germany showed that 5.5% of women with GDM were diagnosed with diabetes already three months after giving birth (Schäfer-Graf et al., 2009). About 10 years later, 14-40% of women became diabetic (Albareda 2003, Feig 2008, Lauenborg 2004), 50% after 15 years (Ryan 2001) and 73% after 25 years (O'Sullivan , 1989). In the long term, GDM can lead to other metabolic abnormalities (Ballas et al 2012) with major implications for public health, especially in the context of resource-poor health systems. Children born to mothers with GDM have a higher risk becoming obese and diabetic (Yogev and Visser, 2009). In study from the US, the risk for children born macrosomic for a metabolic syndrome (obesity, dyslipidemia, hyperglycemia, hypertension) at age 11 was doubled (Boney et al., 2005).

In Morocco, classified as a lower middle income country with a total population of 33.8 million, more than 1.67 million people suffer from diabetes (IDF 2015). The prevalence of diabetes in Morocco is 7.7% (IDF 2015) and it is estimated that the number will double in the next 20 years. Although diabetes is more common in urban areas, 34.6% of diabetes cases in the MENA region are already reported from rural areas (IDF 2013).

An estimated 3.4 million women of reproductive age in the MENA region suffer from gestational diabetes or pre-existing diabetes (IDF 2013). A study conducted between 2008-2009 at Rabat University Hospital, reported a prevalence of gestational diabetes of 8.2% (Bouhsain et al., 2014), but national level figures are unknown. The effects of non-communicable diseases on maternal and neonatal health have not yet been widely studied in Morocco, a country that made good progress towards reaching the MDG targets. With a decline in its maternal mortality ratio from 300 in 1990 to 112 per 100,000 live births in 2011 and a current fertility rate of 2.2 (HCP 2014), the goal to improve

maternal health is increasingly focussing on reducing morbidity that is associated with pregnancy, childbirth and the postpartum period.

Although recommendations on best practice and decision algorithms for the detection of gestational diabetes exist, recommendations in the different guidelines differ. The Moroccan Action Plan for the Reduction of Maternal and Neonatal Mortality recommends a fasting blood glucose in health centres and in reference hospitals free of charge as part of the recommended blood tests during ANC (Ministry of Health 2011).

During a workshop held at the National School of Public Health (ENSP) in October 2014 with the participation of different partners working in the field of diabetes and maternal health in Morocco, it was concluded that knowledge of GDM in Morocco is very limited and that there is a need to fill the knowledge gap regarding detection and management of GDM in Morocco. Therefore a cross-sectional study using a mixed method design was conducted having the following objectives; to describe the current situation of screening and management of diabetes during pregnancy (gestational diabetes and pre-existing diabetes) in the Marrakech-El Haouz region; to identify issues related to screening and management of GDM; to describe the existing knowledge and understand the perceptions about gestational diabetes in pregnant women and in health care professionals and to explore what information pregnant women receive about GDM during ANC. Data was collected in 15 health centres, referral hospitals (provincial, regional and University hospitals), and in three private practices.

The results of this situation analysis indicated that the situation of screening is deficient in Morocco. Although fasting blood glucose forms part of the blood tests prescribed at ANC, blood glucose in the public sector is only taken in hospital laboratories by appointment and following a quota rule per day. This means that women sometimes have to wait two to four weeks to get an appointment for the blood tests that include fasting blood glucose. Oral glucose tolerance tests are rarely performed and only on specific request. If pregnant women are diagnosed with a hyperglycemia, they are referred to a gynecologist and/or endocrinologist. Getting appointments with public sector endocrinologists can take long because these are very busy with an already high number of diabetics. As a result, women often revert to the private sector. So far, all women with gestational diabetes who require medication are being treated with insulin, despite international recommendations for using as alternative the oral antidiabetic drug, metformin, as first-line therapy. Considering that the time for a therapeutic intervention in pregnancy is limited to only a few months, any delay in the detection of GDM and in the initiation of its treatment hinders a timely management that could reduce GDM related complications for mother and her newborn.

Proposed research

The national guidelines on detection and the management of GDM are being revised according to the latest international recommendations to present a GDM screening and management approach that can be integrated into ANC at the first level of care. This implementation research will evaluate the implementation of a country-adapted screening and management strategy developed in collaboration with the Ministry of

Health which is based on the most recent FIGO Consensus Guidelines and adapted to the findings of the situation analysis on GDM. This study therefore intends to evaluate the feasibility of a universal GDM screening and initial management approach through primary health centres, to assess facilitators and challenges of integrating this model into the existing primary health care system and to analyse its cost-effectiveness.
The study contains two main components:

A. Capacity building of primary health care providers in GDM screening and follow-up of women affected by GDM;
B. Effectiveness-implementation research to evaluate this decentralized approach of GDM screening and management in a sample of primary care health structures using a cluster randomized controlled trial design.

Objectives and hypotheses

General objective

This study aims to evaluate the effectiveness of the implementation of a strategy that has been adapted to the Moroccan context regarding the detection of gestational diabetes and its management in primary health care facilities to improve access of pregnant women to screening and thus contribute to a reduction of maternal and neonatal morbidity in Morocco.

Hypotheses

Detection and management of gestational diabetes in Morocco is fragmented between different levels of care. This presents a major obstacle for the timely detection of gestational diabetes and renders monitoring of patients with GDM difficult.

The detection of gestational diabetes in all pregnant women (universal detection) and the management of patients with an uncomplicated GDM at the primary level of care will:

- Increase the number of women detected with a DG;
- Facilitate early care provision to women with GDM;
- Improve the knowledge of health care providers and women about GDM and ensure better compliance of affected women with the recommended treatment;
- Reduce complications of GDM in newborns and the number of macrosomes;
- Reduce the incidence of diabetes (mothers and children) in the long term (not assessed).

Specific objectives

1) Improve universal access to DG detection and follow-up;
2) Strengthen the skills of providers of primary health care facilities regarding the detection, initial management and follow-up of patients with GDM;
3) Measure the prevalence of gestational diabetes in the population served by the intervention facilities;

4) Explore the effects of a decentralized management of GDM on maternal weight gain / blood glucose during pregnancy and measure the proportion of complications around childbirth;
5) Investigate the effects of a decentralized management of GDM on birthweight and the condition of the newborn at birth (Apgar);
6) Examine the effects of a decentralized management on lifestyle changes of GDM affected women;
7) Compare the perceptions of women followed by the different protocols regarding their treatment and the quality of care received;
8) Measure the proportion of women retested for diabetes postpartum;
9) Evaluate adherence to the guidelines in the intervention structures;
10) Evaluate the acceptability of screening and follow-up at the primary level of care by clients and providers and understand facilitators and challenges for their daily practice;
11) Evaluate the costs of the intervention for the health system and for pregnant women;
12) Generate evidence of best practices and existing challenges.

Methods

Research design

We will conduct a hybrid effectiveness-implementation research using a cluster randomized controlled trial design. This design allows us to evaluate both clinical effectiveness of the proposed strategy and its implementation at the first level of care (Curran et al. al., 2012).

Duration

The study will start after approval by the Ethics Committees (Ethics Committee for Biomedical Research at Mohammed V University, Rabat, IRB (IMT) and UZA Antwerp). The research is planned to take place over a period of 12 months.

Study sites

The study will take place in the prefecture of Marrakech and the province of Al-Haouz (Figure 1). The Marrakech Safi region is the second largest region in Morocco in terms of its number of maternal deaths identified by the maternal death surveillance system and one of the priority areas of the Ministry of Health (MS 2013). The prefecture of Marrakech, an essentially urban area is fairly representative of any other urban setting. The province of Al Haouz, a rural and mountainous province where access to health care is difficult, represents the difficulties encountered in the rural provinces of Morocco. The region of Al Haouz is also well known by the researchers who documented pre- and postpartum morbidity.

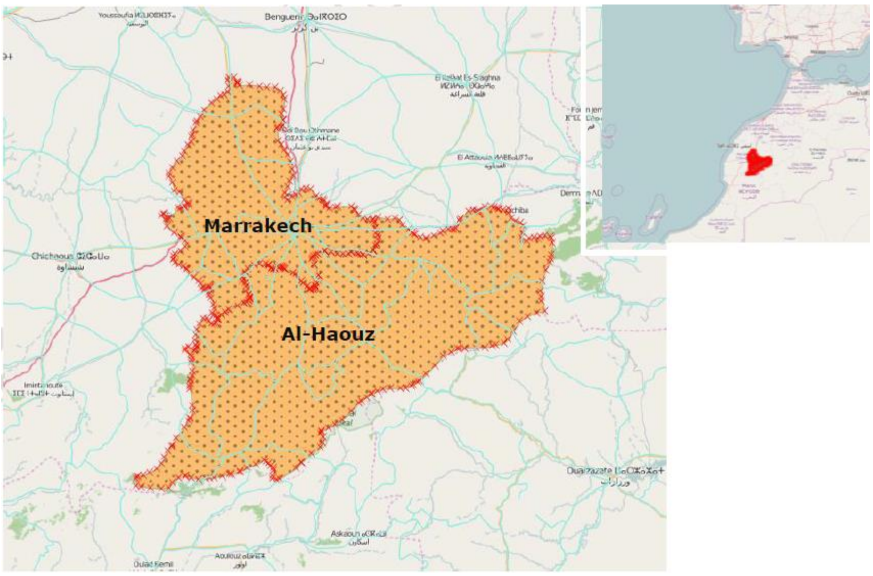

Figure 1: Prefecture of Marrakech (urban) and province of Al Haouz (rural)

We will conduct the research in Marrakech and Al Haouz in 20 primary health care facilities whose number of antenatal consultations is equal to or greater than 30 consultations (new cases) per month. Simple random sampling will be applied for the selection of the facilities (Table 1). We will include 10 facilities in the intervention group (5 in Marrakech and 5 in Al Haouz), in which the new detection and management model will be implemented. Ten structures (5 in Marrakech and 5 in Al Haouz) will serve as controls.

Tableau 1: Primary health care facilities in Marrakech and Al Haouz with 30 or more ANC consultations / month on average (SIAAP, 2014).

**Al Haouz**				**Marrakech**			
Health facility	*Type*	*NC ANC/yr 2014*	*NC ANC /mth on average*	*Health facility*	*Type*	*NC ANC/yr 2014*	*NC ANC/mth on average*
**OURIKA**	CSRA	566	47	**SID ZOUINE**	CSCA	559	47
**ASNI**	CSRA	692	58	**OUDAYA***	CSCA	735	61
**TAMESLOHT***	CSRA	774	64	**KETTARA***	CSCA	358	30
**TOUAMA**	CSRA	504	42	**ELMASSIRA***	CSUA	728	61
**TAKERKOUST**	CSRA	663	55	**Y. BEN TACHFINE***	CSUA	653	54
**AIT OURIR***	CSUA	659	55	**MHAMID***	CSU	807	67
**AMIZMIZ***	CSUA	1190	99	**MAATALAH**	CSU	406	34
**TAZART***	CSC	508	42	**AKIODE**	CSU	404	34
**AIT AADEL**	CSC	556	55	**KOBBA**	CSU	597	50
**TAHANOUT***	CSU	621	51	**OASIS**	CSU	458	38
				**ECHOUHADA**	CSU	407	34
				**AIN ITTI**	CSU	470	39
				**DAR TOUNSI**	CSU	408	34
				**TAMNSOURT**	CSU	454	38
				**SAADA***	CSC	410	34
				**HARBIL**	CSC	359	30
				**C/S 44**	CSC	551	46

	**O-S-B**	CSC	557	46

CSRA: Rural health centre with maternity; CSUA: Urban health centre with maternity; CSCA: Community health centre with maternity; CSR: Rural health centre; CSU: Urban health centre; CSC: Community health centres ;
*facilities included in situation analysis 7-12.2015.

Population

We intend to screen all pregnant women who attend ANC in the intervention facilities included in this research. All women diagnosed with a gestational diabetes will be recruited over a period of 8 to 12 weeks in each group. The follow-up of the patients will be assured until 6 weeks after the last woman recruited in the study delivered.

The inclusion criteria are as follows:

- All pregnant women attending ANC in the health facilities included (screening)
- Women diagnosed with a gestational diabetes at the study sites (follow-up);
- Informed consent to participate in this study.

The exclusion criteria are as follows:

- Woman already known being diabetic type 1 or 2
- Women who did not consent to participate in this study.

Sampling strategy

In the international literature (Langer et al., 2005, Landon et al., 2009) a difference in birth weight between women with untreated DG versus those women treated is highlighted. Langer (Langer et al., 2005) demonstrated in a case-control study including 1100 cases and 555 controls that was conducted between 1990 and 1999 in the United States a difference in birth weight of 330 grams between newborns of untreated women with GDM compared to newborns of women treated for GDM. For the calculation of our sample we assumed that the initial treatment based on the new protocols by the providers at the level of the health centres will generate a birthweight difference with lower birthweights in the intervention group compared to the birthweight of newborns of women managed according to standard practice (control).

Taking into account the cluster effect when calculating the size of our sample (bilateral test of comparison of means) we assume that birthweight of newborns in the "control" group will be 3700g (SD +/- 500g) and birthweight in the "intervention" group 3400g (ET +/- 500g). Adjusting for the cluster design with 20 structures included, a power of 80%, an alpha of 0.05 and an intra-class coefficient (rho) of 0.1, we require 75 women diagnosed with a GDM in each arm (intervention and control).

Based on regional data for these health centres (SIAAP, 2014), indicating an average of 48 antenatal consultations in health facilities with 30 or more ANC per month, we calculated to see about 2000 new ANC registrations per month in the 20 health centres

included. Assuming a prevalence of 8.2% (Bouhsain et al., 2014), we would therefore need to include at least 900 pregnant women in each group to detect a gestational diabetes in 75 women per group.

Progress of the different research components

Part A: Capacity strengthening of health care providers in GDM screening and initial management of women affected by GDM.

After validation of the modified protocol on detection and management of GDM (algorithms) and of the Moroccan GDM nutritional guidelines by the scientific committee, we will train providers of the selected health facilities (intervention) in their practical application. A list of the training content is provided in Table 2.

Table 2: Training plan

Training	Invited participants	Content
**Day 1**	Nurses MNH, midwives, medical doctors of the health centres (intervention group)	○ General information GDM, morbidity and consequences for mother and her newborn<br>○ Information et advice regarding study on GDM<br>○ How to measure capillary blood sugar<br>○ How and when to do an OGTT 75g<br>○ Diagnosis of GDM<br>○ How to calibrate a glucometer<br>○ Nutrition: Calculation of caloric requirements and planning of a nutritional therapy<br>○ Information about nutrition with locally available food<br>○ Counseling about nutrition and exercice<br>○ Counseling glucose control<br>○ Counselling about fœtal monitoring (kick chart)<br>○ Post-partum retesting of women affected by GDM<br>○ Documentation
**Day 2**	General practitioners of health centres (intervention group)	○ Medical treatment of GDM : when to start treatment<br>○ Glycemic control<br>○ Interval and content of follow-up<br>○ Monitoring foetal growth<br>○ How to align routine gynaecological follow-up with GDM follow-up<br>○ How to administer metformin/ insulin<br>○ When to consult an endocrinologist<br>○ Referral slip and communication<br>○ Identifying and treatment of a hypoglycaemia (mother and newborn)<br>○ Assure follow-up post-partum and during the life cycle<br>○ Preconception advice for affected women

Part B: Effectiveness-implementation research to pilot and evaluate this new approach in a sample of primary health care facilities in the prefecture of Marrakech and Al-Haouz district.

We will conduct a cluster randomized controlled trial with an intervention and a control group. In the control group (10 facilities -5 in Marrakech and 5 in Al-Haouz), women will be screened and managed according to standard practice, which usually includes a blood

test performed at the laboratory and referral of positively tested women to a specialist for further care.

In the intervention group (10 sites - 5 in Marrakesh and 5 in Al-Haouz) we will pilot the new approach of GDM screening and its initial management at the primary level of care. A capillary fasting blood glucose will be performed at the first prenatal visit already at health centre level. Women will be re-invited at a gestational age of between 24 and 28 weeks for a 75g oral glucose tolerance test (OGTT) that will also be done in the health centres (if the first test was normal).

Glycaemia testing is theoretically available at the health centres (capillary) and is also part of the standard blood tests for pregnant women done in the laboratories using venous blood. The OGTT, however, is currently only conducted on particular command in some laboratories. For this pilot study we will not introduce a new test but decentralize those tests already recommended in the national guidelines (MS, ANAM 2013) and make them more accessible for all pregnant women at the level of the health centres (see decision tree figure 2 as an example before validation)

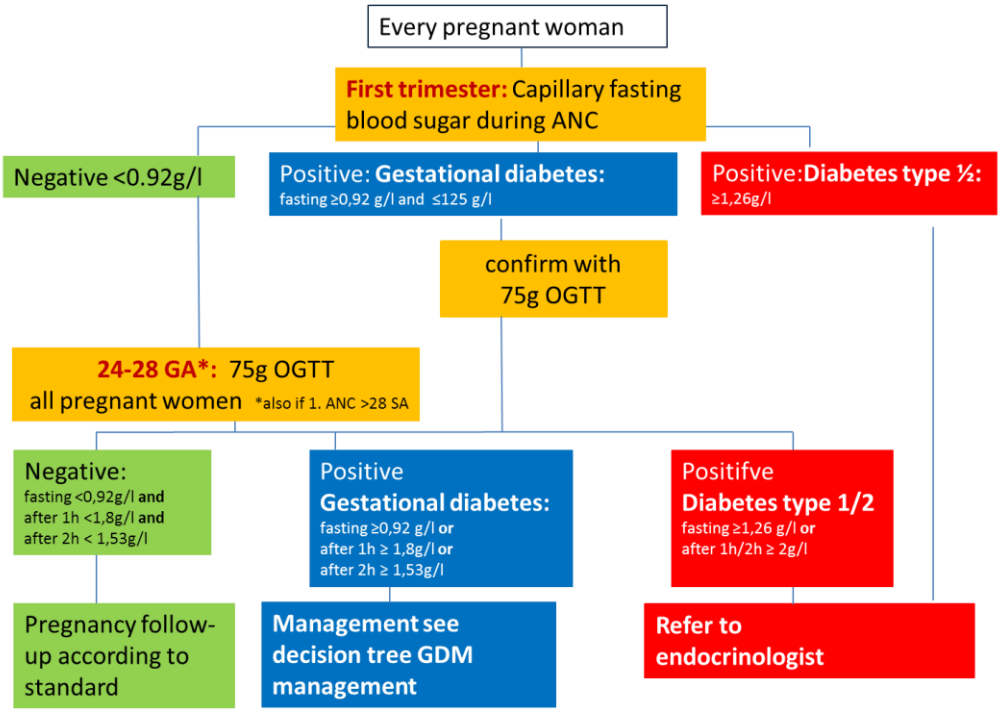

Figure 2: Decision tree example proposed for the universal detection of GDM. Modifed according to Hod et. al (2015) and Indian Ministry of Health and Family Welfare (MOHFW 2014)

At the intervention sites, all pregnant women will be informed during ANC about the importance to screen for gestational diabetes. Those diagnosed with a GDM will be informed about the management according to the new established protocol. We will seek informed consent of all eligible pregnant women with GDM for being included in this study and to be contacted by our investigators during pregnancy and after delivery. All women diagnosed with a GDM will receive locally adapted nutritional advice coupled with information about moderate physical exercise during pregnancy; the management

will be carried out according to the established protocol (algorithm; see figure 3) after its validation by the scientific committee which will also define the follow-up intervals for women diagnosed with a GDM at their health centre. Routine obstetric surveillance will be provided by a gynaecologist according to standard practice. Women with GDM will be asked to be re-tested for diabetes six weeks after delivery (scheduled in combination with the immunization visit at the health centre).

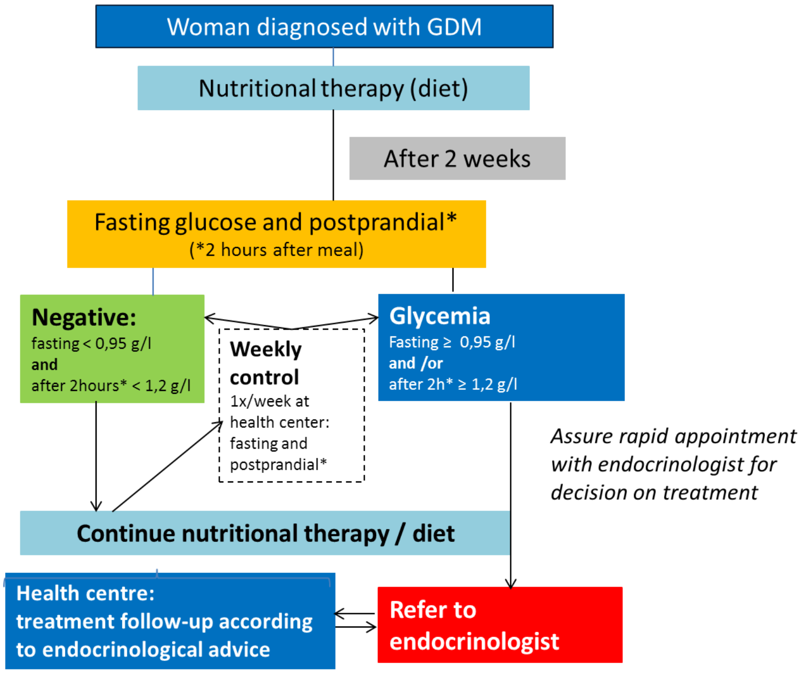

Figure 3: Example of decision tree for the management of women with GDM at the health centre level. Modifed according to Hod et al (2015) and Indian Ministry of Health and Family Welfare (MOHFW 2014)

Evaluation

To evaluate the introduction of GDM detection and management protocols at the health centres, we will collect data in relation to the following objectives:

1. Improve universal access to DG detection and surveillance.
Indicators: (See Table 3)

Table 3: Selection of preliminary indicators

a.	*No. of women tested for GDM with a fasting blood sugar/ No. of women attending ANC (new inscriptions).*	*k.*	*No. of women receiving nutritional therapy only / No. of women followed-up*
b.	*Mean gestational age of women tested with a fasting blood sugar*	*l.*	*Mean gestational age of women tested with an OGTT*
c.	*No of women tested for GDM with a 75g OGTT/ No. of women attending ANC*	*m.*	*No. of women with a GDM receiving medical treatment / No. of women with GDM followed-up*
d.	*No. of women diagnosed with a hyperglycaemia (GDM/DM)/ No. of women tested*	*n.*	*Mean interval (weeks) between diagnosis and treatment initiation*
e.	*Mean gestational age of women diagnosed with a hyperglycaemia*	*o.*	*Mean gestational age at treatment initiation*
f.	*No. of women receiving nutritional therapy*	*p.*	*Interval between beginning of nutritional therapy and medical treatment*
g.	*No. of women referred to an endocrinologist / No. of women diagnosed with a GDM*	*q.*	*No. of women monitored at health centres lost to follow-up / No. of women monitored*
h.	*No. of women with a GDM followed-up at health centre level/ No. of women diagnosed with a GDM*	*r.*	*Mean interval (weeks) between GDM diagnosis and delivery*
i.	*No. of follow-ups per woman with GDM at health centre level / No. of women diagnosed with GDM at the health centre*	*s.*	*Mean interval (weeks) between nutritional therapy and delivery*
j.	*Mean follow-up interval (weeks)*	*t.*	*Mean interval (weeks) between medical treatment and delivery*

2. Strengthen the capacities of providers of primary health care facilities in the detection, initial management and follow-up of patients with GDM.

Indicators:
Pre-and post-test assessing GDM knowledge and practice before and after the training (immediate pre- and post-test and 4 weeks later).

3. Calculate the prevalence of gestational diabetes.

Indicator:
No. of women tested positive for a GDM / No. of women tested for GDM

4.Investigate the effects of management of GDM at health centre level on the woman's weight gain / blood sugar during pregnancy and on complications around childbirth

Indicators:
Mean blood glucose levels during follow-up
Weight at the beginning of pregnancy (BMI);
Evolution of weight during pregnancy (IMC categories);
Mode of delivery;
Complication during childbirth: dystocia, pre-eclampsia, prolonged labour, stillbirth, preterm birth, macrosomia.

5. Investigate the effects of GDM management on birthweight the newborn’s condition at birth.

Indicator:
Birthweight;
APGAR of the newborn (1 'and 5').
Associated morbidity (hypoglycemia, respiratory distress, cardiomyopathy ..)

6. Evaluate the effects of a decentralized management on lifestyle changes of women with GDM.

Indicator:
Level of agreement or disagreement with a lifestyle statement (change of diet, adherence to physical exercise) using Likert scales (Questionnaire for women with GDM after delivery)

7. Analyse the perceptions of women followed-up according to different protocols regarding treatment and quality of care received.

Indicator:
Degree of agreement or disagreement with a statement regardingvtreatment and quality of care using Likert scales (Questionnaire for women with GDM after delivery)

8. Measure the proportion of women re-tested for diabetes in the post-partum period (6 weeks postpartum).

Indicator:
No. women with GDM re-tested postpartum (up to 6 weeks) / No. of women diagnosed with GDM.

9. Evaluate adherence to the algorithms on GDM screening in intervention facilities.

Indicator:
Observation of GDM screening practice (Audit)

10a. Evaluate the acceptability of GDM screening and monitoring at the level of primary health care facilities by pregnant women and to understand facilitators and challenges for women.

Semi-structured interviews with approximately 30 women diagnosed with GDM at the level of the study sites (15 at intervention and 15 at control sites – e.g. each fifth woman included in each group)

10b. Evaluate the acceptability of GDM screening and monitoring by primary level health care providers and explore facilitators and challenges for their daily practice; assess their adherence and ease of use of the new protocol.

Focus groups with health care providers responsible for detecting and monitoring women with GDM in their primary health care facilities.

11) Evaluate the costs of the intervention for the health system and for pregnant women.

Indicators:

Calculation of the costs of a decentralized management of GDM.
For the health service:
- Additional costs and costs of the current application of the strategy
- Amortization of equipment costs;
- Recurrent costs: laboratory tests, expenditure on drugs, time spent on screening and management
For the patients:
- direct costs for women, including expenses for travel, laboratory tests, drugs and equipment

Source: Costs of intervention, structured questionnaire and interviews with women with GDM.

Data collection and tools

After training of providers, pregnant women in different health care facilities will be screened for DG according to the protocols (intervention group: new protocol, control group: standard practice). Women tested positive for GDM will be followed-up according to the protocols (intervention group: new protocol, control group: standard practice). After obtaining informed consent of the women, contact details of each woman diagnosed with GDM will be documented by the health workers so that investigators can contact them later.

Quantitative data

Quantitative data at health centre level will be collected by the investigators who will visit the structure monthly and who will remain in close contact with the personnel involved in the detection and monitoring of women. Data will be extracted using a data extraction form and entered into an Excel database on a monthly basis. The principal investigator will be in contact with the field team and supervise the data collection.

Data collected at the facility level includes monthly information on the number of women screened and followed-up and the material used. To collect individual information on the women screened / followed-up we will add a data sheet on GDM to be placed in the health record of each woman included in this study to enable the health worker to directly document information on this sheet. Through a survey post-partum, women's perceptions of the quality of care, implications of GDM on their lifestyle and expenses will be evaluated. Surveys will be conducted by the investigators 6 to 8 weeks postpartum either at the woman's home, or at a location chosen by the woman at an appropriate time, or by telephone if a visit is not feasible.

Data on the results of pre-and post-test to assess knowledge and practice will be collected using a structured questionnaire distributed among the participants before and after the training.

Qualitative data

For the qualitative data collection semi-structured interviews will be done with a sample of the women included in this study after they gave birth to assess their perceptions about GDM detection at the level of their health centre, their perceptions about the follow-up,

the quality of care received, changes in quality of life after diagnosis and the costs involved. We will also conduct two focus-group discussions (FGDs) with the providers in charge of GDM screening and monitoring of women with GDM in the intervention facilities to better understand their perceptions and experience with this new model, its advantages and its challenges for their daily practice (three to six months after the start of the intervention). FGDs will take place at the Health Directorates in Marrakech and Al Haouz.

In addition, we intend to interview 10 key informants (the number will depend on the saturation of the information received) to obtain information on their opinion regarding this new approach and the potential to scale it up.

We will conduct observations of ANC sessions in facilities included in the study (5 CPN per centre) to assess the time spent for GDM screening and management (used for cost calculation). In the intervention structures we will also evaluate adherence to the screening algorithms.

All interviews and focus group discussions will be conducted at an appropriate moment for the participants and transportation costs to attend the interview will be reimbursed. The interview guides and FGD guides are provisional since they will be adapted following an iterative process of qualitative methodology.

Interviews with providers will be conducted in French, and with women in Arabic / Berber by trained investigators. They will be digitally recorded after obtaining permission by the interviewees.

Table 4: Data to collect

**CONTROL**		**INTERVENTION**	
5 urban health centers	5 rural health centers	5 urban health centers	5 rural health centers
		Pre- and post-tests providers	
Monthly facility data		Monthly facility data	
Individual data (75+)		Individual data (75+)	
Observation ANC consultations (5 ANC per centre)		Observation ANC consultations (5 ANC per centre)	
Survey (75+)<br>In-depth interviews with subsample of women		Survey (75+)<br>In-depth interviews with subsample of women	
Focus groups with health care providers;<br>(1 Marrakech, 1 Al Haouz)		Focus groups with health care providers;<br>(1 Marrakech, 1 Al Haouz)	
Key-informant interviews (national /regional; 10)			

Data analysis

Qualitative data analysis

The interviews and focus groups will be recorded, translated and transcribed and data organized using NVIVO10 software. A thematic content analysis will be carried out and new emerging themes arising during the interviews or focus groups will be integrated integrated (mixed approach: inductive and deductive).

Quantitative data analysis

Quantitative data will be double-entered and analysed with Stata 13IC software (College Station Texas USA) / SPSS Version 21. Descriptive statistics will be performed based on the specific measurements of each set of data collected.

Through the combination of quantitative and qualitative data using a diversity of data collection methods, we will be able to triangulate the data and can thus strengthen the internal validity of the study results.

Monitoring, supervision and data quality control

All quantitative data will be collected by two investigators trained in data collection and collection will be supervised during the trial by the principal investigator who will be on site. Interviews with key informants and focus groups with providers will be conducted by the principal investigator in French assisted by one of the local investigators. Surveys and structured interviews with women will be conducted in Arabic or Berber. We hope to reduce the refusal to participate in the study by a patient -friendly attitude and a clear explanation of the importance of the study. The number of refusals will be quantified and the reasons for the refusal documented. The quality of data entry will be assured by controlled double entry.

Quality assurance

To ensure the quality of the project, a scientific committee will be set up to monitor the project and ensure its implementation. The scientific committee will be involved in: (1) adapting the Moroccan guidelines to the latest FIGO recommendations; (2) development of training tools for health professionals; (3) monitoring the context-specific model of gestational diabetes care. Regular quarterly meetings will be held between the project team and the advisory working group to provide updates on the process. The Principal Investigators (BU / BA) will closely supervise the implementation of the project and be in direct communication with the investigators and the facility managers during the implementation of the project.

Ethical considerations

This study will be subject to the review and formal approval of the IRB / ITM, UZA in Antwerp and the Ethics Committee of the University of Rabat. None of the participants will be recruited and no activity related to the study started before obtaining the written approval of these organizations. The study will be conducted in accordance with the principles set out in the Declaration of Helsinki, all applicable regulations and established international scientific standards.

Obtaining informed consent

In our study we will make sure that all women included in the study and interviewees at the level of the health facilities provided their informed consent. We will make sure that participants recruited are well aware of: i) their participation is voluntary; (ii) that they can ask any questions they wish and that they will receive understandable and useful

answers in deciding whether or not to participate; iii) that they may withdraw at any time during the study and withdraw data concerning them at any time and without consequences for them. After reading the briefing note, the interviewer invites those who agreed to participate in the research to read the informed consent form and to sign or leave their fingerprints (depending on their level of education.). If a respondent is unable to read or write, a signature from a witness to the informed consent will be obtained. The witness will be chosen by the participant.

Data safety

Access to the data will be restricted to the researchers involved in the study. Investigators will ensure anonymity and confidentiality of the information collected, by limiting access to this information. Under no circumstances will investigators disclose the information collected during the investigation. The data will be kept under lock and key and electronic data files will be password protected. The use of the data in any other research will be subject to a standardized procedure for verifying the objectives of the research and only in agreement of the ethics committees. We are aware that we will access medical records with personal data. Investigators and all others having access to the data will sign a statement to respect absolute confidentiality of the data and to protect the anonymity of the participants.

Risks

The GDM screening and management algorithms will be validated by national experts before being applied in the facilities. The blood glucose tests will be carried out as part of the routine service delivery according to approved standards and validated by a scientific committee at the national level. We will not test new therapeutic products or diagnostics that are not yet practiced in Morocco. In the intervention structures we will apply a glucose tolerance test (OGTT 75g) for diagnosing GDM, the gold standard test recommended in national (MS, ANAM 2013) and international guidelines on GDM detection (Hod et al, 2015). This test can cause nausea and vomiting because of the hyper-osmolarity of the glucose solution, a complication that can affect 3.7% of women (Mohan et al., 2014). Measuring blood glucose by capillary sampling during GDM screening and follow-up may be associated with moderate pain caused by the finger prick and may be accompanied by a burning sensation.

Advantages

Participation in this study has the advantage that women in the intervention facilities will benefit from a screening test that corresponds to the latest international consensus guidelines and will be carried out at their local health centre. As we only include 10 health centres in the intervention group, a limited number of women will benefit from the new GDM screening and management approach at health centre level. However, as the Ministry of Health is involved in the implementation and evaluation of the project, a potential scaling up of this new approach to the national level resulting from this pilot project might be of larger future benefit for pregnant women and their newborns.

Use of study data in other research

The use of the data in other research will be subject to a standard procedure for verifying the objectives of the research and after agreement of the ethics committee (s).

Dissemination of results

A study report will be written after finalizing data analysis and submitted to the Ministry of Health and its different directorates and to the organizations providing financial support to the study. The dissemination and publication of the study will be done by the principal investigators at IMT and ENSP and findings published in an international peer-reviewed journal.

The results of this study will also be presented at the level of Marrakech-Safi region during a meeting with the relevant official representatives (Regional Directorate of Health, health delegations and representatives of local facilities and civil society associations) in the presence of the researchers.

Activity chart

	M1	M2	M3	M4	M5	M6	M7	M8	M9	M10	M11	M12	M13	M14	M15	M16	M17	M18
Protocol development	■	■																
Finalising new GDM protocol	■	■																
Development of tools for data collection		■	■															
Protocol submission to IRB/ Ethics Committees		■	■	■														
Development of training material			■	■	■													
Recrutement of research assistants					■													
Training of research assistants					■													
Piloting tools						■												
Training of providers ; pre/post-test						■												
Inclusion of women into study							■	■										
Observation ANC							■	■										
FGD health care providers									■	■								
Key informant interviews										■	■							
Surveys and interviews with women with GDM											■	■	■	■	■			
Supervision and data collection							■	■	■	■	■	■	■	■	■			
Data analyses									■	■	■	■	■	■	■	■	■	
Dissemination of findings																	■	■
Final report																		■

Budget

No.		€
**1.**	**Training**	**2500 €**
1.1	Training 2 days for 40 providers	
**2.**	**Consumables**	**5500 €**
2.1	Teststrips for glucometers : 6000 (25 for 100MAD)	2400€
2.2	Specific testing material (1000 x 75g OGTT, lancettes, etc.)	2500€
2.3	Glucometers (plasmacalibrated) x 15 (à 400 MAD)	600€
**3.**	**Photocopies, training material**	**1000 €**
**4.**	**Communication**	**1000 €**
4.1	Telephone credit providers / research assistants during 10 months	22x 50 MAD/month
**5.**	**Dissemination meetings**	**1800€**
**6.**	**Personnel**	**15200€**
6.1	Research assistants (2) (transport costs)	450MAD/dx13d/x9
6.2	Travel expenses PI during 10 months	3800€
6.3	Local data collection 50x160	800€
6.4	Transcription	1000 €
**7.**	**Transport participants interviews (70 participants)**	**1000€**
**8.**	**Other (5%)**	**1200 €**
	**TOTAL**	**29400 €**

References

Albareda, M., Caballero, A., Badell, G., Piquer, S., Ortiz, A., DeLeiva, A., Corocoy, R.(2003) Diabetes and abnormal glucose tolerance in women with previous gestational diabetes. Diabetes Care, 26, p. 1199-1205.

Bellamy L., Casas, J.P., Hingorani, A., Williams, D. (2009). Type 2 diabetes mellitus after gestational diabetes: a systematic review and meta-analysis Lancet; 373: 1773–79

Boney, C.M., Verma, A., Tucker, R., & Vohr, B. R. (2005). Metabolic syndrome in childhood: association with birth weight, maternal obesity, and gestational diabetes mellitus. Pediatrics, 115(3), p. e290-e296.

Bouhsain, S., El Kochri, S., Babahabib, M.A., Hafidi, M.H., Bouaiti, E., et al. (2014). Comparing two screening policies of gestational diabetes mellitus: The Mohammed V Training Military Hospital of Rabat (Morocco). *Gynecologie, obstetrique & fertilite*. pii: S1297-9589(13)00270-1. doi:10.1016/j.gyobfe.2013.09.006.

Curran, G. M., Bauer, M., Mittman, B., Pyne, J. M., & Stetler, C. (2012). Effectiveness-implementation hybrid designs: combining elements of clinical effectiveness and implementation research to enhance public health impact. Medical care, 50(3), 217.

El Amrani, F.Z. (2012) ; Diabète et grossesse. Thèse No. 38. Université Mohammed V : Faculté de Médicine et de Pharmacie : Rabat, Maroc.

Fadl, H., Oestlund, I., Magnuson, A., Hanson, U. (2010) Maternal and neonatal outcomes and time trends of gestational diabetes in Sweden from 1991 to 2003. Diabetic Medicine, 27, p. 103-107.

Feig, D.S., Zinman, B., Wang, X., Hux, J.E. (2008). Risk of development of diabetes mellitus after diagnosis of gestational diabetes. *Canadian Medical Association Journal;* **179**(3): 229-234.

FID (2015). *Diabetes Atlas Sixth Edition*. International Diabetes Federation.Available from: www.idf.org/diabetesatlas [Accessed 9.3.2015]

Hod M, Kapur A, Sacks DA, Hadar E, Agarwal M, Di Renzo GC et al. (2015). Management of hyperglycemia during pregnancy. Int J Gynaecol Obstet;131(S3):S190-200

Hyperglycemia and Adverse Pregnancy Outcome (HAPO) Study Cooperative Research Group. (2008) Hyperglycemia and Adverse Pregnancy Outcomes. New England Medical Journal, 358, p. 1991-2002.

Landon, M. B., Spong, C. Y., Thom, E., Carpenter, M. W., Ramin, S. M., Casey, B., et al. (2009). A multicenter, randomized trial of treatment for mild gestational diabetes. New England Journal of Medicine, 361(14), 1339-1348.

Langer, O., Yogev, Y., Most, O., & Xenakis, E. M. (2005). Gestational diabetes: the consequences of not treating. American journal of obstetrics and gynecology, 192(4), 989-997.

Lauenborg, J., et al. (2004). Increasing Incidence of Diabetes After Gestational Diabetes A long-term follow-up in a Danish population. *Diabetes Care;* **27**(5): 1194-1199.

Ministère de la Santé (2012). ENPSF 2011. Rabat, Morocco: Ministère de la Santé.

Ministère de la Santé. Enquête confidentielle sur les décès maternels au Maroc - 2010, Rabat: Ministère de la Santé du Royaume du Maroc, 2013. 42 pages.

MS, ANAM 2013 Recommandations de Bonnes Pratiques Médicales Affection Longue Durée ALD 6 (Selon l'Arrêté Ministériel) Diabète de type 2 (CIM 10/E11): Rabat: ANAM

MOHFW(2014). National Guidelines for Diagnosis & Management of Gestational Diabetes Mellitus, India. Ministry of Health and Family Welfare, Maternal Health Division.New Delhi: UNICEF

O'Sullivan, J. (1989) The Boston Gestational Diabetes Studies: Review and Perspectives. In: Sutherland, H., Stowers, J., Pearon, D. (eds.). Carbohydrate metabolism in pregnancy and the newborn IV. London: Springer.

Ryan, E. (2001) What is Gestational Diabetes? In: Gerstein, H.C., Haynes, R.B. (eds..) Evidence-Based Diabetes Care. Hamilton-London: BC Decker Inc.

Schaefer-Graf, U., Klavehn, S., Hartmann, R., Kleinwechter, H., Demandt, N., Sorger, M., Kjos, S., Vetter, K., Abou-Dakn, M. (2009) How do we reduce the number of missed postpartum diabetes in women with recent gestational diabetes? Diabetes Care, 32, p. 1960-1964.

SIAAP (2014). Système de routine, Marrakech-Tensift-Al Haouz, Morocco: SIAPP.

Syed, M., Javed, H. , Yakoob, M.Y., Bhutta, Z.A.et al. (2011). Effect of screening and management of diabetes during pregnancy on stillbirths. *BMC Public Health*; **11**(Suppl 3): S2.

WHO (2013). Diabetes factsheet [online]. Available at: http://www.who.int/mediacentre/factsheets/fs312/en/. [Accessed 17 September 2015].

Yogev, Y. and G. H. Visser (2009). Obesity, gestational diabetes and pregnancy outcome. *Seminars in Fetal and Neonatal Medicine*; 14 (2): 77–84
